# The effects of cash transfers and vouchers on the use and quality of maternity care services: A systematic review

**DOI:** 10.1371/journal.pone.0173068

**Published:** 2017-03-22

**Authors:** Benjamin M. Hunter, Sean Harrison, Anayda Portela, Debra Bick

**Affiliations:** 1 Department of International Development, King’s College London, London, United Kingdom; 2 School of Social and Community Medicine, University of Bristol, Bristol, United Kingdom; 3 Department of Maternal, Newborn, Child and Adolescent Health, World Health Organization, Geneva, Switzerland; 4 Florence Nightingale Faculty of Nursing and Midwifery, King’s College London, London, United Kingdom; Stellenbosch University, SOUTH AFRICA

## Abstract

**Background:**

Cash transfers and vouchers are forms of ‘demand-side financing’ that have been widely used to promote maternal and newborn health in low- and middle-income countries during the last 15 years.

**Methods:**

This systematic review consolidates evidence from seven published systematic reviews on the effects of different types of cash transfers and vouchers on the use and quality of maternity care services, and updates the systematic searches to June 2015 using the Joanna Briggs Institute approach for systematic reviewing. The review protocol for this update was registered with PROSPERO (CRD42015020637).

**Results:**

Data from 51 studies (15 more than previous reviews) and 22 cash transfer and voucher programmes suggest that approaches tied to service use (either via payment conditionalities or vouchers for selected services) can increase use of antenatal care, use of a skilled attendant at birth and in the case of vouchers, postnatal care too. The strongest evidence of positive effect was for conditional cash transfers and uptake of antenatal care, and for vouchers for maternity care services and birth with a skilled birth attendant. However, effects appear to be shaped by a complex set of social and healthcare system barriers and facilitators. Studies have typically focused on an initial programme period, usually two or three years after initiation, and many lack a counterfactual comparison with supply-side investment. There are few studies to indicate that programmes have led to improvements in quality of maternity care or maternal and newborn health outcomes.

**Conclusion:**

Future research should use multiple intervention arms to compare cost-effectiveness with similar investment in public services, and should look beyond short- to medium-term service utilisation by examining programme costs, longer-term effects on service utilisation and health outcomes, and the equity of those effects.

## Introduction

Prospective users of health services in many countries face financial costs for transport and treatment and opportunity costs of receiving care, and such costs are well-documented barriers for the uptake of maternity care services [1–3]. “Demand-side” financing in the health sector was introduced to promote health by offsetting some associated financial costs or by increasing household income or providing financial incentives to increase healthy behaviours. Five types of demand-side financing have been used to promote maternal and newborn health [4]:

- *Conditional cash transfers* aim to increase utilisation of maternity care services by making regular payments to households conditional on attendance at community meetings and/or uptake of government health and education services.
- *Unconditional cash transfers* are similar regular payments but in the absence of conditions for service utilisation, aim to alleviate the effects of poverty on maternal health.
- *Short-term cash payments to offset costs* are typically retrospective payments made at healthcare facilities to those who attend for care.
- *Vouchers for maternity care services* aim to reduce the cost of maternity care services at point of use.
- *Vouchers for ‘merit’ goods* aim to reduce the cost of buying goods (such as food or insecticide-treated nets) that promote maternal and newborn health.

To date there have been seven published systematic reviews of evidence on the impact of aspects of demand-side financing on maternal health (Table 1). These concluded that demand-side financing increases uptake of maternity care services shorter-term but evidence of population level uptake over longer periods of time was limited. Few studies collated data for more than two to three years, and therefore conclusions could not be drawn about longer-term effects, and evidence did not enable impacts to be assessed on maternal and newborn health, quality of care, or cost-effectiveness. Murray *et al*. 2014 [4] also noted that most studies included in their review were unable to assess impacts on health outcomes due to small sample sizes.

**Table 1:** Systematic reviews of demand-side financing.

Systematic review	Types of demand-side financing included
Lagarde *et al*. 2007 [5]	Conditional cash transfers
Bellows *et al*. 2011 [6]	Vouchers for maternity care services
Meyer *et al*. 2011 [7]	Vouchers for maternity care services
Murray *et al*. 2012; 2014 [4,8]	Conditional cash transfers, unconditional cash transfers, short-term payments to offset costs, vouchers for maternity care services and vouchers for merit goods
Brody *et al*. 2013 [9]	Vouchers for maternity care services
Glassman *et al*. 2013 [10]	Conditional cash transfers and short-term payments to offset costs
Gopalan *et al*. 2014 [11]	Conditional cash transfers, unconditional cash transfers, short-term payments to offset costs and vouchers for maternity care services

Recent systematic reviews completed literature searches during or before June 2012 [4,8,10,11]. We conducted a systematic review to consolidate the previous reviews and included additional studies (up to June 2015), in order to answer two questions: 1) what are the effects of different demand-side financing interventions on maternity care service utilisation and maternal and newborn health outcomes in low- and middle-income countries, and 2) what is the evidence on the cost-effectiveness of demand-side financing to increase utilisation of maternity care services and improve maternal and newborn health outcomes?

In this paper, we present the results related to the impact on maternity care services utilization, equity and quality of care. The complete report that details the information for all the outcomes is available upon request from the corresponding author and the World Health Organization.

## Methods

This systematic review is an update of the *effectiveness* component of an earlier review of 72 articles (36 quantitative and 36 qualitative studies) registered with the Joanna Briggs Institute (registration number 000592) [4,8]. The review protocol for this update (S1 Appendix) was registered with PROSPERO (CRD42015020637), and is reported according to PRISMA guidelines (S2 Appendix).

The population of interest were women of all ages who were pregnant or within 42 days of giving birth. Interventions of interest were any programme that incorporated demand-side financing as a method to increase the utilisation of maternity care goods and services expected to have an impact on maternal and newborn health outcomes. The review only considered studies relevant to populations in low- and middle-income countries defined as such by the World Bank at the time study data were collected.

The primary review outcomes were measures of utilisation of maternity care services, including antenatal, birth and postnatal care. Other outcomes included equity, quality of care as defined by study authors (including use of life-saving commodities) and cost-effectiveness, maternal mortality and morbidity, perinatal and neonatal mortality and morbidity. Uptake of family planning methods and services was not considered.

The review only included experimental and non-experimental (observational) study designs. An earlier version of the review (by Murray *et al*.) conducted searches for 1990 to June 2012. This review included the quantitative studies retrieved by Murray *et al*., those identified and included in other systematic reviews outlined in Table 1 and new studies that met inclusion criteria published between July 2012 and June 2015. There were no limits on length of follow-up, language of publication or publication status.

The following databases and e-journal services were searched: Applied Social Sciences Index and Abstracts, ArticleFirst, British Development Library Services, EBSCO Host (includes CINAHL and MEDLINE), Cochrane Central Register of Controlled Trials, EconLit, Electronic Collections Online, HealthSource: Nursing/Academic Edition, International Bibliography of the Social Sciences, Latin-American and Caribbean Center on Health Sciences Information (LILACS), Sage Journals Online, ScienceDirect, SCOPUS, Social Policy and Practice, Social Services Abstracts, Sociological Abstracts, SpringerLink, Web of Knowledge, and Wiley Online Library. The search for unpublished studies included: Archives of relevant governmental and non-governmental organisations and development banks, Intute, Nexis UK, Mednar, ProQuest Dissertations and Theses, Qual Page, Scirus, and WorldWideScience.org.

A search using a matrix of 32 keywords and index terms was undertaken across all included information sources, and a sample search strategy for SCOPUS is shown below:

1. (“child benefit” or “demand side financing” or “demand-side financing” or “family allowance” OR “food stamp” or “maternity allowance” or “maternity benefit”)
2. (“cash transfer” or “monetary transfer” or “output-based aid” or “results-based financing” or “reimbursement mechanism” or “voucher” or “incentive”)
3. (“abortion” or “antenatal” or “birth” or “infant” or “matern$” or “midwi$” or “neonat$” or “obstetric” or “perinatal” or “postpartum” or “postnatal” or “pregnan$”).ti,ab
4. (“cost” or “cost-effectiv$” or “cost-utility” or “health service utili$” or “morbidity” or “mortality”).ti,ab
5. 3 or 4
6. 1 and 5
7. 2 and 3
8. 6 or 7 [Limit to: Publication Year 2012–2015]

The title and abstracts of retrieved papers were screened against the following inclusion criteria: date of publication, population of interest, intervention, context and outcome. For those that appeared to meet inclusion criteria, a full-text paper was retrieved. Full-text papers were then screened against the same criteria, and reference lists (including those of previous systematic reviews mentioned above) examined for relevant studies. Studies were excluded if the methods were insufficiently described to allow assessment, if they lacked a comparator (time-point or group), or if they lacked any testing for statistical significance.

Standardised critical appraisal instruments from the Joanna Briggs Institute were used to assess papers for methodological validity and risk of bias (JBI-MAStARI for studies on health outcomes, service use and quality of care, and JBI-ACTUARI for studies on costs and cost-effectiveness) [12]. Two independent reviewers assessed each paper using a set of 9–11 questions and conferred. Questions included the representativeness of the study sample, strategies for dealing with confounding factors and choice and measurement of outcomes.

The review team considered it important to give an overall quality rating to individual studies, a feature not included in the JBI approach, in order to facilitate interpretation of study findings. A three point rating system (low-, medium- or high-quality) was adopted, similar to that used to assess study bias by the Effective Public Health Practice Project (EPHPP) quality assessment tool for quantitative studies [13]. In the current review the quality rating was based on the reviewers’ overall assessment of each study. High-quality studies were those with appropriate sampling strategies and analytical methods which collated data from a period spanning more than three years after programme introduction to allow adequate time for longer-term effects to be assessed. Disagreements were resolved through discussion, or with a third reviewer.

Data were extracted by two independent reviewers (BMH and DB) using standardised data extraction tools from the Joanna Briggs Institute (JBI-MAStARI for data on health outcomes, service use or quality of care, and JBI-ACTUARI for cost and cost-effectiveness data) [12]. Extracted data included specific details about the populations, interventions, context, study methods, risk of bias and outcomes of significance to the review questions.

Data are presented in narrative form including tables to aid in data presentation where appropriate. Meta-analyses could not be undertaken due to the heterogeneity of interventions, settings, study designs and outcome measures. Albatross plots [14] were created to provide a graphical overview of the data for interventions with more than five data points for an outcome. Albatross plots are a scatter plot of p-values against the total number of individuals in each study. Small p-values from negative associations appear at the left of the plot, small p-values from positive associations at the right, and studies with null results towards the middle. The plot allows p-values to be interpreted in the context of the study sample size; effect contours show a standardised effect size (expressed as relative risk—RR) for a given p-value and study size, providing an indication of the overall magnitude of any association. We estimated an overall magnitude of association from these contours, but this should be interpreted cautiously.

Exact p-values were extracted from all studies where possible, however studies only reporting that the p-value was not significant (e.g. p>0.05), and for which there was no means of calculating the exact p-value, were displayed as a line for the valid range of p-values (e.g. 0.05 to 1). Equally, for studies stating only that a p-value was significant at a certain threshold (e.g. p<0.05), that p-value was assumed to be equal to the threshold (e.g. p = 0.05). Relative risks from effect contours were converted to attributable risks, assuming a control group uptake of 50%, to aid in interpretation. Studies were excluded from the plots if there was insufficient information, usually because the number of study participants was not reported, and instances of this are highlighted at appropriate points in the results section. Where studies reported subgroups rather than an overall result, the results from subgroups were taken, so each study may provide more than one point on a plot.

In this paper we only present results relating to the impact of demand-side financing interventions on maternity care services utilisation, equity and quality of care. The ability of demand-side financing interventions to impact on indicators of maternal and newborn morbidity and mortality is unclear with little robust evidence that any of the five types of demand-side financing considered improved these outcomes. Such outcomes are harder to measure than utilisation. Included studies were generally methodologically poor with intervention outcomes mainly considered in the short-term and with some contradictory findings.

## Results

**Fig 1:**
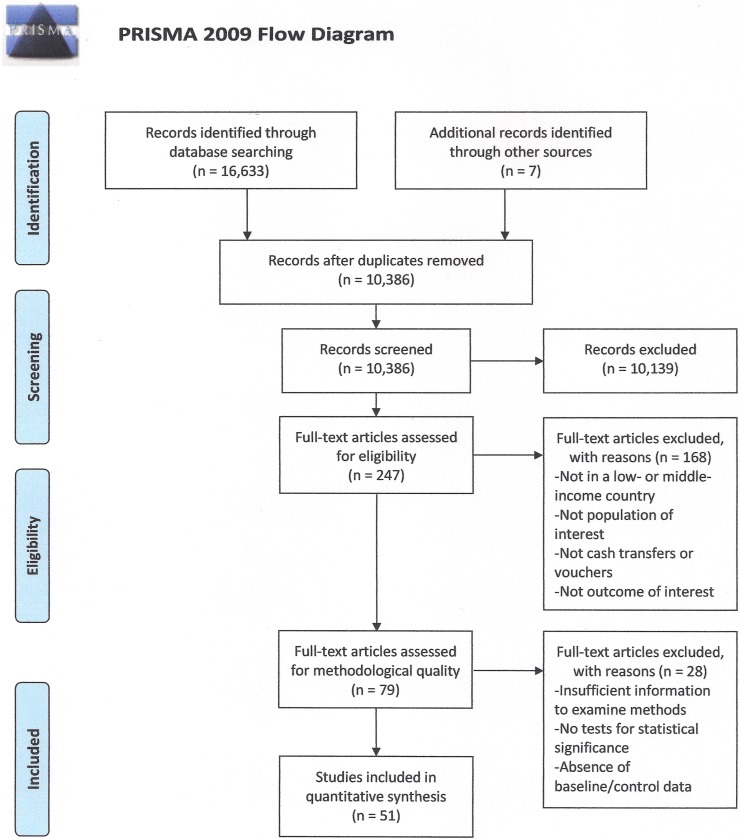
Systematic review flowchart. *From*: Moher D, Liberati A, Tetzlaff J, Altman DG, The PRISMA Group (2009). *P*referred *R*eporting *I*tems for *S*ystematic Reviews and *M*eta-*A*nalyses: The PRISMA Statement. PLoS Med 6(7): e1000097. doi:10.1371/journal.pmed1000097. **For more information, visit** www.prisma-statement.org.

The systematic searches identified 10,380 individual records. After titles and abstracts were screened, 241 full text articles were retrieved (Fig 1). Additionally, seven articles were identified in the reference lists of articles and other published systematic reviews. Of these, 79 quantitative studies were carried forward for critical appraisal. Twenty-eight did not meet minimum requirements for methodological quality.

The review of effectiveness included 51 studies relating to 22 demand-side financing programmes: seven on conditional cash transfers, one on unconditional cash transfers, four on short-term cash payments to offset costs of access, nine on vouchers for maternity care services, and one on vouchers for merit goods. (See S3 and S4 appendices for critical appraisal results and assessment of bias for each study, and study characteristics tables). Compared to the earlier systematic review by Murray *et al*. 2014 [4], an additional 15 studies were included in the current review.

The study designs reported varied, including six cluster randomised controlled trials (RCTs); one quasi-experimental design and 41 observational studies: 18 observational studies used before-and-after studies with comparison areas, four before-and-after studies without comparison areas, 11 cross-sectional studies with comparison areas, and eight used panel data as part of a retrospective area study. The five studies that examined data on costs, cost-effectiveness or cost-utility (two of which are included above as they also included data on maternity care service utilisation) were cross-sectional studies without comparison areas (but with comparison time points).

Data sources ranged from national registers to small household surveys. The methodological quality of the included studies varied. The most common weaknesses in study methods included small sample sizes, follow-up periods less than three years, lack of adjustment to take account of potential confounding factors or secular trends. As observational studies may not have sufficient statistical power to detect ‘true’ effects, findings should be interpreted with caution.

The findings are presented below according to type of demand-side financing.

### Conditional cash transfers

Programmes that included conditional cash transfers typically focused on reducing poverty by improving the health and education of children. Many included attendance at antenatal care as a conditionality for payments, and some aimed to improve the health and wellbeing of pregnant women. Sixteen studies on conditional cash transfers were included, relating to seven programmes (see Table 2):

- Bolsa Familia in Brazil [15,16],
- Comunidades Solidarias Rurales in El Salvador [17],
- Mi Familia Progresa in Guatemala [18].
- Programa de Asignación Familia in Honduras [19],
- Program Keluarga Harapan in Indonesia [20,21],
- Prospera in Mexico [22–29], and
- Plan de Atención Nacional a la Emergencia Social (PANES) in Uruguay [30],

**Table 2:** Details of conditional cash transfer programmes included in the systematic review.

Programme/ Period of implementation	Included studies, quality, sample size and year data collected	Maternal and newborn health entitlements	Supply-side components	Details of any changes to programme design	Source of funding
Bolsa Familia, Brazil/ 2003-present	Guanais (2013)—high quality: 54,213 women during 1998–2010 [15]; Shei (2013)—high quality: national data during 1998–2008 [16]	Households receive, on average, 170 real (USD 75) monthly (conditional on ANC visits for pregnant women in a household)	No, but took place alongside expansion of the Family Health Program	None identified	National government
Comunidades Solidarias Rurales, El Salvador/2005-present	De Brauw and Peterman (2011)—medium quality: 269 and 287 households at baseline and follow-up during 2008 [17]	USD 30, monthly (conditional on ANC visits for pregnant women)	No, but took place alongside investments in health system infrastructure in intervention areas	Phased roll-out, beginning with poorest areas	National government
Mi Familia Progresa, Guatemala/2008-present	Gutierrez (2011)—medium quality: 4,563 households during 2009 and 2010 [18]	150 quetzales (USD 15), monthly (conditional on ANC visits for pregnant women)	No	None identified	National government
Programa de asignación Familiar, Honduras/1990-present	Morris (2004)—medium quality: 11,002 households during 2000 and 2002 [19]	Vouchers worth 55 lempiras (USD 4), monthly (conditional on ANC visits for pregnant women)	No, but some intervention areas were supposed to receive funds for improving healthcare infrastructure. A study on the programme indicated that this did not take place (Morris *et al*. 2004)	Programme design altered in 1998 to increase value of vouchers and revise eligibility criteria	National government
Program Keluarga Harapan, Indonesia/2008-present	Alatas *et al*. (2011)—medium quality: 14,987 and 14,922 women at baseline and follow-up during 2007 and 2009 [20]; Triyana (2012)—medium quality: 14,987 and 14,922 women at baseline and follow-up during 2007 and 2009 [21]	250,000 rupiah (USD 28) per quarter to households with a pregnant or lactating mother (conditional on 4 x ANC, SBA and 2 x PNC for mothers and newborns)	No	Piloted in five provinces	National government
Prospera (previously Oportunidades), Mexico/1997-present	Barber and Gertler (2008)—medium quality: 840 women during 2003 [23]; Barber and Gertler (2009)—medium quality: 892 women during 2003 [24]; Barber (2010)—medium quality: 979 women during 2003 [22]; Barham (2011)—high quality: 19,421 women during 1992–2001 [25]; Hernandez Prado *et al*. (2004a)—high quality: 2,445 municipalities during 1995–2002 [26]; Hernandez Prado *et al*. (2004b)—medium quality: 29,041 and 7,802 women at baseline and follow-up during 1998–2000 and 2003 [27]; Sosa-Rubai *et al*. (2011)—high quality: 5,051 women during 2007 [28]; Urquieta *et al*. (2009)—medium quality: 2,790 women during 1998 and 2000 [29]	180 pesos (USD 17), monthly (conditional on five ANC visits and attendance at health education talks)	No	Phased roll-out and payment size increased periodically	National government
Plan de Atención Nacional a la Emergencia Social (PANES), Uruguay& 2005–2007	Amarante *et al*. (2011)—medium quality: 67,863 women during 2003–2007 [30]	1,360 pesos (USD 55), monthly (conditional on regular ANC in 2007)	No	Payment size adjusted for inflation	National government

Notes: ANC—antenatal care, SBA—birth with a skilled birth attendant, PNC—postnatal care

Most of the studies were assessed as of medium quality. Study designs utilised survey and register/facility data with large sample sizes and (where applicable) took advantage of a phased programme roll-out. Methodological issues included potential leakage of vouchers or payments to control groups and short periods of time before follow-up surveys were conducted. Methodological weaknesses were inherent in aggregation of datasets from diverse areas in national studies and many studies did not disaggregate data using markers of known social inequalities such as wealth quintile and geographic location.

#### Conditional cash transfers and antenatal care

Eight studies examined outcomes of conditional cash transfers on uptake of antenatal care (Table A1 in S5 Appendix). Evidence of effectiveness compared to control areas (‘usual care’) was found in one cluster RCT (n = 11,002) of the Programa de Asignación Familia in Honduras [19]. Similarly, evidence of better outcomes in intervention areas compared to control areas was found in controlled before-and-after studies of Guatemala’s Mi Familia Progresa (n = 4,563), Indonesia’s Program Keluarga Harapan (n = 29,909) and Plan de Atención Nacional a la Emergencia Social in Uruguay (n = 67,863) [18,20,30].

The controlled before-and-after study by Hernandez Prado *et al*. [27] of Mexico’s Prospera programme found uptake of antenatal care varied depending on when the programme was introduced in a particular area. One cluster RCT (n = 840 women) and one cross-sectional study (n = 5,051) of the Prospera programme found no evidence of effect [23,28]. A controlled before-and-after study (n = 556) of El Salvador’s Comunidades Solidarias Rurales also found no evidence of effect on uptake of antenatal care [17].

**Fig 2:**
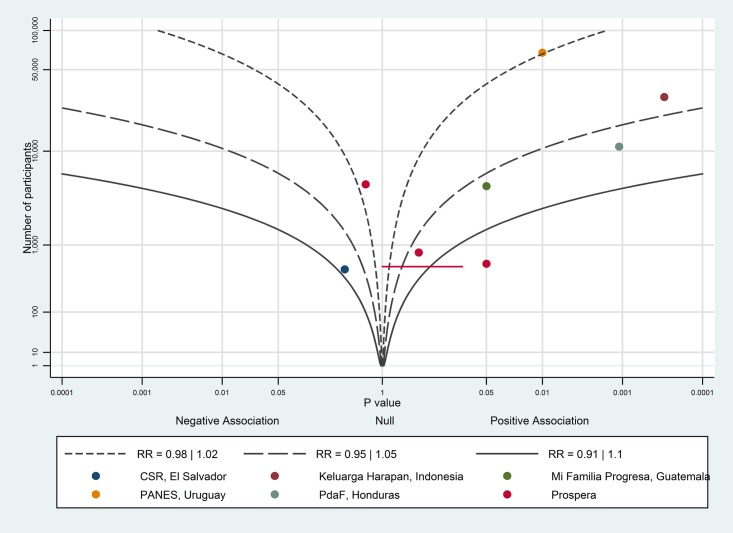
Albatross plot for effect of conditional cash transfer programmes on uptake of antenatal care. CSR—Comunidades Solidarias Rurales; PANES—Plan de Atención Nacional a la Emergencia Social; PdAF—Programa de Asignación Familiar.

The albatross plot for uptake of antenatal care is shown in Fig 2. Seven of the nine data points showed a positive association between conditional cash transfers and antenatal care uptake. Most of the studies were around the RR contour of 1.05, corresponding to about a 2–3 percentage point increase in uptake of antenatal care. However, the studies on Mi Familia Progresa, Programa de Asignación Familia and Program Keluarga Harapan all fall around the 1.05 RR contour, and have percentage point increases of 7.2–18.7, therefore a 10 percentage point increase might be a more reasonable estimate of the effect of conditional cash transfers on uptake of antenatal care; we considered this to be a large effect.

#### Conditional cash transfers and birth with a skilled birth attendant

Seven studies examined the effect of conditional cash transfers on births with a skilled birth attendant (Table A2 in S5 Appendix). A cross-sectional study (n = 5,051) of Mexico’s Prospera programme found increased use of a skilled birth attendant in intervention compared to control areas [28], however another study of the same programme which used a controlled before-and-after design (n = 36,843) reported that births with a skilled attendant varied between rural and urban areas depending on when the programme was introduced [27]. Another controlled before-and-after study of the Prospera programme (n = 2,790) found no evidence of effect [29].

A controlled before-and-after study (n = 556) of El Salvador’s Comunidades Solidarias Rurales found the programme had some effect on births with a skilled birth attendant in intervention compared to control areas, but differences were not statistically significant [17]. A controlled before-and-after study (n = 29,909) of Indonesia’s Program Keluarga Harapan found no evidence of effect [20], however another study (which used the same data but different analyses) reported that the programme did increase births with a skilled attendant compared to control areas [21]. A controlled before-and-after study (n = 67,863) on Uruguay’s Plan de Atención Nacional a la Emergencia Social found no evidence of effect [30].

**Fig 3:**
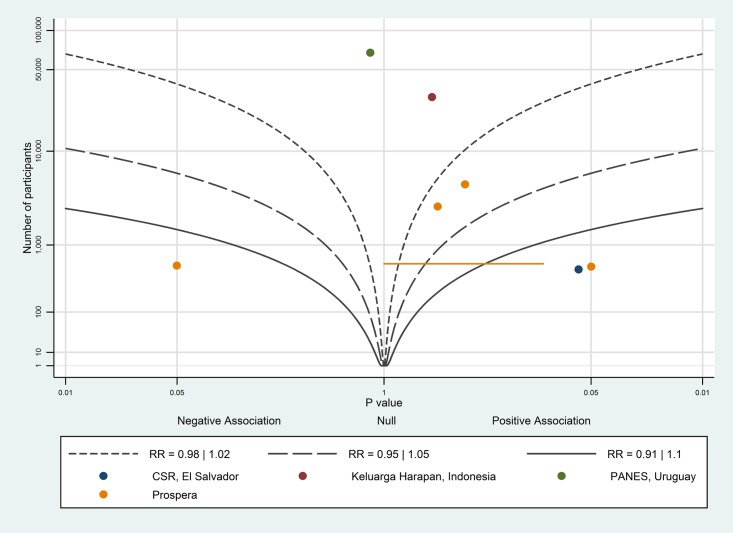
Albatross plot for effect of conditional cash transfer programmes on births with a skilled birth attendant. CSR—Comunidades Solidarias Rurales; PANES—Plan de Atención Nacional a la Emergencia Social.

The albatross plot for births with a skilled birth attendant is shown in Fig 3. Seven of the nine data points showed a positive association, however the studies showed variation in the magnitude of effect indicating there may be meaningful differences between them. Additionally, the larger studies [20,30] showed no effect. Therefore, overall, the evidence showed only a very small positive effect of conditional cash transfers on birth with a skilled birth attendant.

#### Conditional cash transfers and births in a healthcare facility

Three studies, all of which used a controlled before and after design, examined the effect of conditional cash transfers on births in a healthcare facility (Table 3). A study (n = 556) of El Salvador’s Comunidades Solidarias Rurales found some evidence that births in a healthcare facility increased when compared to control areas [17]. A study (n = 4,563) of Guatemala’s Mi Familia Progresa found some evidence that the programme increased births in small public healthcare facilities and decreased births in public hospitals, although this was only reported in early intervention areas [18]. The third study included in this section (n = 29,909) of Indonesia’s Program Keluarga Harapan found no evidence of effect [20].

**Table 3:** Effect of conditional cash transfers on births attended at a healthcare facility.

Study	Study data	Effect	95% confidence interval, standard error or p-value
*Program Keluarga Harapan*, *Indonesia (2008-present)*			
Alatas *et al*. (2011)	2007, 2009	No evidence of effect	p>0.05
*Comunidades Solidarias Rurales*, *El Salvador (2005-present)*			
De Brauw and Peterman (2011)	2008	22.8 percentage point increase	se: 5.2 (p<0.01)
*Mi Familia Progresa*, *Guatemala (2008-present)*			
Gutierrez *et al*. (2011)	2009, 2010	5.0 percentage point decrease in birth in public hospitals in early intervention areas	p<0.1
		3.0 percentage point increase in births in health posts in early intervention areas	p<0.1
		No evidence of effect in late-intervention areas	p>0.1

#### Conditional cash transfers and birth with a skilled birth attendant or birth in a healthcare facility in case of complications

Two studies examined the effect of Prospera on caesarean section rates in Mexico. One cluster RCT (n = 979) indicated that the caesarean section rate among recipients of Prospera was 14.5%, which was 5.1 percentage points higher than non-recipients (p = 0.05) [22]. A controlled before-and-after study (n = 36,843) found no evidence of effect on caesarean section rate (p>0.05), which was noted to be 19.5% among recipients in urban areas, 12.6% among recipients in early intervention rural areas and 16.9% in late intervention areas [27].

#### Conditional cash transfers and postnatal care

Three studies examined the effect of conditional cash transfers on uptake of postnatal care for women and their newborns (Table 4). A controlled before-and-after study (n = 29,909) of Indonesia’s Program Keluarga Harapan found an increase in the proportion of women and newborns who had two or more postnatal contacts compared to women and newborns in the control group, but no evidence of an overall increase in the mean number of visits [20]. No evidence of effect on uptake of any postnatal care for mothers and newborns was found by a cluster RCT (n = 11,002) of the Programa de Asignación Familia in Honduras [19], or a controlled before-and-after study (n = 556) of El Salvador’s Comunidades Solidarias Rurales [17].

**Table 4:** Effect of conditional cash transfers on uptake of postnatal care for mothers and newborns.

Study	Study data	Effect	95% confidence interval, standard error or p-value
*Comunidades Solidarias Rurales*, *El Salvador (2005-present)*			
De Brauw and Peterman (2011)	2008	No evidence of effect^1^	p>0.1
*Programa de Asignación Familia*, *Honduras (1990-present)*			
Morris *et al*. (2004)	2000, 2002	No evidence of effect^1^	p>0.05
*Program Keluarga Harapan*, *Indonesia (2008-present)*			
Alatas *et al*. (2011)	2007, 2009	No evidence of effect^2^	p>0.05
		7.0 percentage point increase^3^	se: 0.3 (p<0.05)

Notes.

^1^ any postnatal care

^2^ mean number of visits

^3^ two or more postnatal visits

#### Conditional cash transfers and quality of care

Only two included studies considered the effect of conditional cash transfers on aspects of quality of care. Both studies defined ‘quality’ as the number of procedures undertaken during antenatal contacts. Barber and Gertler [24] reported results from a cluster RCT study (n = 892 women) that measured receipt of 13 antenatal care components in Mexico such as history-taking and diagnostics, physical examination, prevention and case management. They found that recipients of Prospera programme payments received 12.2% more antenatal procedures than non-recipients (p<0.001).

Triyana [21] developed an antenatal quality index based on receipt of diagnostic procedures, information on pregnancy complications, vaccination and iron pills. Their controlled before-and-after study (n = 29,909 women) found recipients of Program Keluarga Harapan payments received 16.7 percentage points more antenatal procedures than non-recipients (p<0.05).

#### Costs, cost-effectiveness and cost-utility of conditional cash transfers

Not measured by included studies.

### Unconditional cash transfers

One study examined the effect of Zambia’s Child Grant Programme, an unconditional cash transfer programme, on maternal and newborn health outcomes [31]. Like many conditional cash transfers, the Zambian Child Grant Programme (2010-present) aims to reduce poverty by improving the welfare of children and payments are distributed regularly to women living in districts with highest rates of child mortality and morbidity. The programme, funded by the Zambian Government, offers recipients 60 kwacha (USD 12) every two months.

Handa *et al*.’s [31] cluster RCT study used data from two household surveys. Baseline data were collected in 2010 and a follow-up survey was conducted in 2012. Handa *et al*. found no evidence of effect of the Child Grant Programme on uptake of antenatal care or on birth with a skilled birth attendant. The study also found no evidence of effect on receipt of specific procedures during antenatal care (receipt of testing and counselling for HIV, tetanus vaccination and malaria treatment).

### Short-term cash payments to offset costs

Short-term cash payments have been used in some countries to offset the costs of accessing maternity care services. Unlike conditional and unconditional cash transfer programmes which aim to reduce poverty by regularly distributing payments to households, short-term cash payments are made retrospectively and on a small, defined number of occasions. Conditionalities for receipt of payments typically include birth in a healthcare facility and may include attendance at antenatal care and postnatal care for mothers and newborns. Thirteen studies on short-term cash payment programmes, relating to four programmes were included (Table 5):

- Janani Suraksha Yojana in India [32–40],
- Safe Delivery Incentive Programme in Nepal [41,42],
- CHIMACA programme in China [43], and
- SURE-P programme in Nigeria [44].

**Table 5:** Details of short-term cash payment programmes included in the systematic review.

Programme	Included studies, quality and sample size	Maternal and newborn health entitlements	Supply-side components	Period of programme implementation	Details of any changes to programme design	Source of funding
CHIMACA programme, China	Hemminki *et al*. (2013)—medium quality: 592 women during 2008–2009 [43]	Payment of up to 20 renminbi (USD 3) for women who used ANC	No	2007–2009	None identified	European Commission
Janani Suraksha Yojana, India	Amudhan *et al*. (2013)—medium quality: 7,796 women during 2006–2010 [32]; Carvalho *et al*. (2014)—medium quality: 23,924 women during 2007–2009 [33]; Joshi and Sivaram (2014)—medium quality: 425,708 women during 2002–2004 and 2007–2009 [34]; Lim *et al*. (2010)—medium quality: 182,869 women during 2002–2004 and 2007–2009 [35]; Mazumdar *et al*. (2012)—medium quality: 344,903 women during 2002–2004 and 2007–2009 [36]; Purohit *et al*. (2014)—low quality: 424 women during 2011 [37]; Randive *et al*. (2013)—medium quality: unknown sample size during 2005–2010 and 2010–2011 [38]; Santhya *et al*. (2011)—medium quality: 4,770 women during 2009 and 2010 [39]; Vora *et al*. (2012)—medium quality: 2,267 women during 2007–2009 [40]	Up to 1,400 rupees (USD 32) in one payment, given after FB	No, but part of the broader National Rural Health Mission which invested in healthcare infrastructure	2006-present	None identified	National government
Safe Delivery Incentive Programme, Nepal	Powell-Jackson *et al*. (2009)—medium quality: 14,799 women during 2001–2007 [45]; Powell-Jackson and Hanson (2012)—medium quality: 5,901 women during 2008 [42]	Up to 1,500 rupees (USD 23) in one payment (conditional on FB)	Incentive payment to a health worker who attends a birth	2005-present	Removed parity restrictions; expanded participating healthcare facilities to include private sector	National government
Subsidy Reinvestment and Empowerment Programme (SURE-P), Nigeria	Okoli *et al*. (2014)—medium quality: 20,133 women during 2013–2014 [44]	Up to 5,000 naira (USD 30) in seven payments after receipt of maternity care services (conditional on 4 x ANC, SBA and PNC for mothers and newborns)	Part of the SURE-P programme, which includes supply-side investment in intervention areas	2013-present	None identified	National government

Notes: ANC—antenatal care, FB—birth in a healthcare facility, SBA—birth with a skilled birth attendant, PNC—postnatal care

The included studies on short-term cash payment programmes were assessed to be of medium quality. Most had large sample sizes however findings are based on follow-up data typically collected only one or two years after programme launch. Many studies from India used the same national survey datasets with the assumption that women who reported receipt of any payment in the survey had received a payment as part of the Janani Suraksha Yojana programme.

#### Short-term payments and antenatal care

Nine studies examined the effect of short-term cash payments on uptake of antenatal care (Table A3 in S5 Appendix). One retrospective area study (n = 14,799 women) of Nepal’s Safe Delivery Incentive Programme found an increase in the mean number of antenatal visits [41]. Three controlled before-and-after studies (sample sizes ranging from 4,770 women to 1,340,427 households) and one cross-sectional study (n = 424 women) of India’s Janani Suraksha Yojana found evidence that the programme increased the proportion of women receiving three or more antenatal visits compared to women who were not programme-recipients [35–37,39]. One controlled before-and-after study (n = 425,708 women) and one cross-sectional study (n = 2,267 women) found no differences in uptake of antenatal care [34,40]. A retrospective area study (n = 20,133 women) of Nigeria’s SURE-P programme found no increase in receipt of any antenatal care [44] and a cluster RCT (n = 592 women) on the CHIMACA programme in China found no evidence of effect on number of antenatal contacts women received [43].

Disaggregated data on Janani Suraksha Yojana presented by Lim *et al*. [35] showed that the programme had a greater effect on the proportion of women receiving three or more antenatal visits in high-focus states (where payments are larger and are made irrespective of income or parity) than in non-high-focus states.

**Fig 4:**
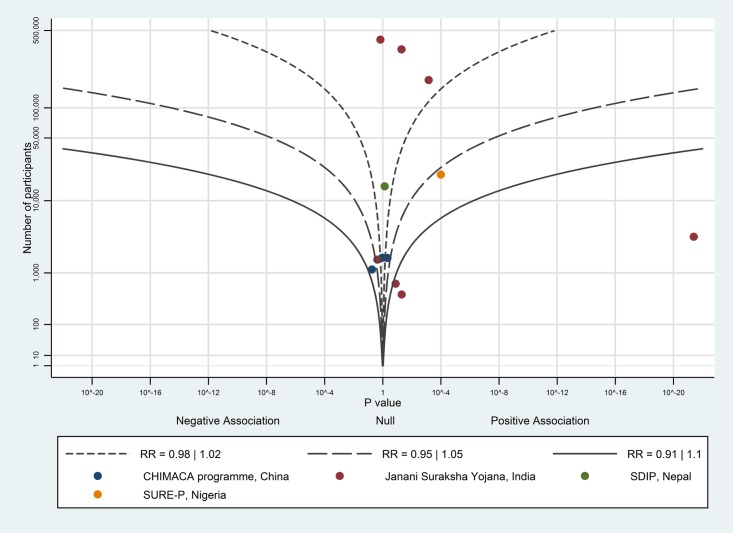
Albatross plot for effect of short-term cash payments on uptake of antenatal care. SDIP—Safe Delivery Incentive Programme.

The albatross plot of short-term payments on uptake of antenatal care is shown in Fig 4. Although eight of the 12 data points showed a positive effect, the studies showed no particular trend along any contour. With the exception of Santhya et al. [39], which showed a very large positive effect (odds ratio of 2.2), the data points tended to fall around the null, possibly favouring a positive association. This suggests a very limited (but possibly positive) effect of short-term payments on uptake of antenatal care.

#### Short-term payments and birth with a skilled birth attendant

Seven studies considered effects of short-term cash payment programmes on births with a skilled birth attendant (Table A4 in S5 Appendix). Four controlled before-and-after studies (using sample sizes ranging from 4,770 women to 1,340,427 households) on Janani Suraksha Yojana in India found evidence that the programme increased births with a skilled birth attendant among recipients compared to non-recipients [34–36,39]. Two studies of Nepal’s Safe Delivery Incentive Programme (one cross-sectional study with a sample size of 5,901, and one retrospective area study with 14,799) found a positive benefit on outcomes assessed compared to control areas [41,42]. No evidence of effect was found by a retrospective area study (n = 20,133) on Nigeria’s SURE-P programme [44].

One of the studies on Janani Suraksha Yojana, using a controlled before-and-after design (n = 1,340,427 households) disaggregated data by type of state (high-focus states as defined by the National Health Mission with low rates of births in healthcare facilities where payments are larger and made irrespective of income or parity, vs. non-high-focus) and found that the programme’s impact on use of skilled birth attendants was greater in high-focus states [35]. Another controlled before-and-after study (n = 4,770 women) found that the impact of Janani Suraksha on use of skilled birth attendants was similar regardless of whether women lived in an urban or rural area [39].

A controlled before-and-after study (n = 5,901) of Nepal’s Safe Delivery Incentive Programme conducted additional analyses to examine heterogeneity in programme effects and found no association between household wealth and programme effect, and that higher payment size relative to costs (as payment size varied according to terrain type) increased the programme’s effect [42]. They also found that the programme was associated with better quality of care. This was measured using data collected in a survey of health providers, and a scoring system according to whether a healthcare facility had performed any of the following in the preceding three months: administered parenteral antibiotics, oxytocics, and/or anticonvulsants; performed manual removal of placenta and/or retained products; performed assisted vaginal delivery and/or caesarean section; given a blood transfusion; referred a woman by ambulance, and/or provided 24 hour maternity care services).

**Fig 5:**
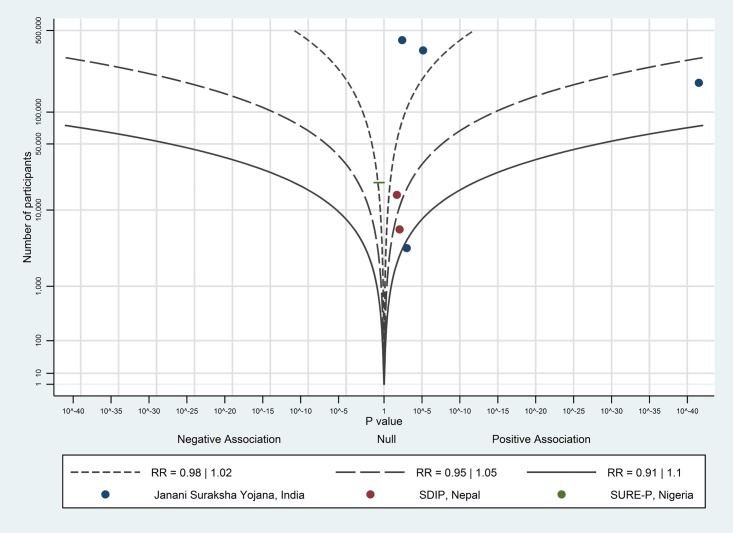
Albatross plot for effect of short-term cash payments on births with a skilled birth attendant. SDIP—Safe Delivery Incentive Programme.

The albatross plot of short-term payments on birth with a skilled birth attendant is shown in Fig 5. Six of the seven data points showed a positive effect; the likely magnitude of effect is around the 1.05 RR contour, corresponding to a 2–3 percentage point increase on birth with a skilled birth attendant; we considered this to be a small effect.

#### Short-term payments and births in healthcare facilities

Eight studies examined the effect of short-term cash payments on whether births took place in healthcare facilities (Table A5 in S5 Appendix). A quasi-experimental study (n = 7,796) and four controlled before-and-after studies (sample sizes ranging from 4,770 women to 1,340,427 households) found evidence that India’s Janani Suraksha Yojana increased births in healthcare facilities [32,35,36,38,39] although a later cross-sectional study (n = 2,267) reported that the programme increased births at healthcare facilities in one Indian state, but not in another state [40]. A retrospective area study (n = 14,799) of Nepal’s Safe Delivery Incentive Programme found no impact on place of birth [41], however a cross-sectional study (n = 5,901) found evidence that the programme increased births at healthcare facilities among women who were aware of the programme, compared to those who were not [42].

Disaggregated data from the controlled before-and-after study referred to above by Lim *et al*. [35] showed that Janani Suraksha Yojana had a greater effect on increasing births in facilities in high-focus states than in non-high-focus states, with findings corroborated by another controlled before-and-after study (n = 5,903 women) and a quasi-experimental study (n = 7,796 women) [32,36]. The smaller controlled before-and-after study also found that births in healthcare facilities were similar for women regardless of household wealth or ethnicity [36]. Janani Suraksha Yojana’s effect on increasing births at healthcare facilities was similar to the effect on location of births following upgrading a healthcare facility to provide access to free 24-hour care [32].

Another controlled before-and-after study (n = 4,770) disaggregated data by type of geography and found that the effect of Janani Suraksha Yojana on place of birth was similar in rural and urban settings [39].

**Fig 6:**
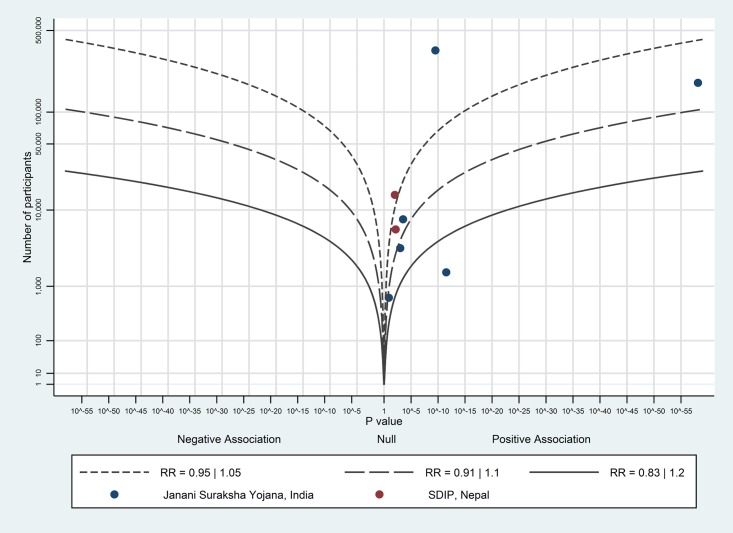
Albatross plot for effect of short-term cash payments on health facility births. SDIP—Safe Delivery Incentive Programme.

The albatross plot of short-term payments on birth in a healthcare facility is shown in Fig 6. One study [38] was not included in the albatross plot as the number of study participants was not available. All eight data points showed a positive effect; the likely magnitude of effect is around the 1.1 RR contour, corresponding to a 5 percentage point increase on birth in a healthcare facility; we considered this to be a moderate effect.

#### Short-term payments and birth with a skilled birth attendant or birth in healthcare facilities in case of complications

Five studies examined the effect of short-term cash payment programmes on caesarean section rates (Table A6 in S5 Appendix). A cluster RCT (n = 592 women) of the CHIMACA programme in China (which offered payments to women if they attended antenatal care), using 104 towns in five counties as clusters, found evidence that the programme increased caesarean section rates, which were 67.1% among intervention clusters and 56.0% among control clusters [43]. A controlled before-and-after study (n = 5,903 women) of India’s Janani Suraksha Yojana found no evidence of effect on caesarean section rates [36], however a cross-sectional study (n = 2,267) found rates increased in one state (where it was 15%) with no evidence of effect in another state [40]. A retrospective area study (n = 14,799) of Nepal’s Safe Delivery Incentive Programme found no evidence of effect on caesarean rates however one cross-sectional study (n = 5,901) found evidence that the programme increased caesarean section rates (from a baseline of 3%) and assisted births compared among women who knew about the programme before giving birth [41].

**Fig 7:**
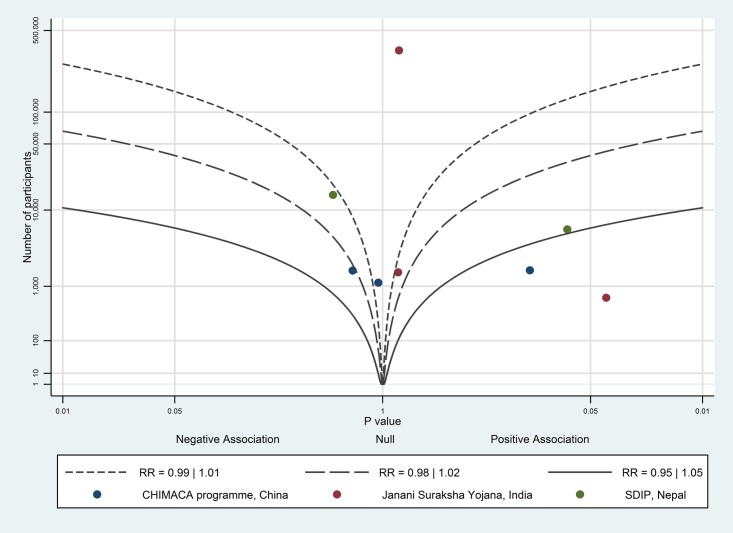
Albatross plot for effect of short-term cash payments on birth with a skilled birth attendant or birth in healthcare facilities in case of complications. SDIP—Safe Delivery Incentive Programme.

The albatross plot of short-term payments on caesarean section rates in case of maternal or foetal complications is shown in Fig 7. Five of the eight data points showed a null association; the three remaining studies showed a small positive association. Overall, there is no evidence to show an effect of short-term payments on caesarean section rates.

#### Short-term payments and postnatal care

Four studies examined the effect of short-term cash payments on receipt of postnatal care for women and newborns (Table A7 in S5 Appendix). A controlled before-and-after study (n = 425,708 women) of India’s Janani Suraksha Yojana reported that the programme was associated with a decrease in the proportion of women and newborns who received a postnatal check within two weeks of the birth [34]. One cross-sectional study (n = 23,924 women) found evidence that the same programme increased receipt of a postnatal check for women and newborns within 24 hours of birth [33] while another cross-sectional study (n = 424 women) also reported that the proportion of women and newborns who received postnatal care increased, but did not explain how postnatal care was defined or measured [37]. A cluster RCT (n = 592 women) of the CHIMACA programme found no evidence of effect on receipt of at least one postnatal contact for mothers and newborns [43].

Two studies examined the effect of short-term cash payments on receipt of maternal postpartum care only. One controlled before-and-after study (n = 4,770 women) found that recipients of India’s Janani Suraksha Yojana were significantly more likely to receive a postpartum check-up within 48 hours of giving birth compared to non-recipients [39]. A cross-sectional study (n = 23,924) found evidence of a 24.8 percentage point increase (compared to non-recipients of the programme) in receipt of a postpartum check-up within 48 hours of giving birth [33].

**Fig 8:**
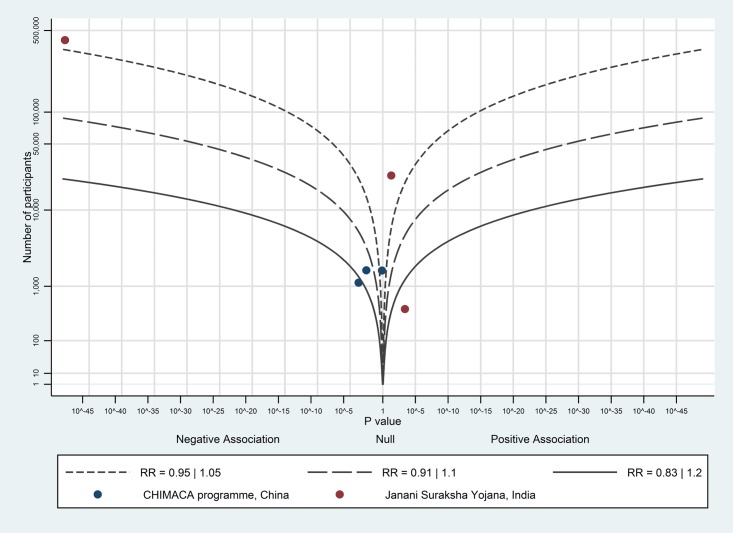
Albatross plot for effect of short-term cash payments on postnatal care for mothers and newborns.

The albatross plot of short-term payments on uptake of postnatal care for women and newborns is shown in Fig 8. Four of the six data points showed a negative effect, including the largest study of 419,156 women [34]. Given this, and that all CHIMACA programme data points came from the same study [43], the evidence showed there is likely a small negative or no association between short-term payments and uptake of postnatal care.

#### Short-term payments and quality of care

Three studies examined the effect of short-term cash payments on quality of care. One controlled before-and-after study (n = 4,770 women—Santhya *et al*. [39]) and one cross-sectional study (n = 424 women—Purohit *et al*. [37]) of Janani Suraksha Yojana reported that more women (p<0.01) were discharged from hospital after at least 24 hours in-patient stay. Santhya *et al*. also examined receipt of specific procedures. While women who received Janani Suraksha Yojana payments were more likely to have been offered information on warning signs and symptoms of adverse health during pregnancy (p<0.05) there were no statistically significant differences in the reporting of receipt of information on postnatal care and neonatal health, clean healthcare facilities, respectful care from members of staff, or use of harmful practices during childbirth (fundal pressure or intra-muscular oxytocin to expedite birth).

The cluster RCT (n = 592 women) by Hemminki *et al*. [43] found evidence that short-term cash payments in the CHIMACA programme increased receipt of some antenatal care procedures (nutrition advice and danger signs and symptoms advice), however no evidence of effect on receipt of other procedures (blood pressure and anaemia tests).

#### Costs, cost-effectiveness and cost-utility of short-term payments

Not measured by the included studies.

### Vouchers for maternity care services

Voucher entitlements typically include labour and birth care in a programme-affiliated (often accredited) healthcare facility and a defined number of antenatal and postnatal care contacts. Some programmes have included access to a combination of public and private providers and some only included access to private sector facilities. The review included 19 studies on vouchers for maternity care services, relating to nine programmes (Table 6):

- Bangladesh’s Maternal Health Voucher Scheme [46–48]
- a pilot voucher scheme in Bangladesh [49],
- a voucher programme in Cambodia [50],
- India’s Chiranjeevi Yojana [51–53]
- Sambhav vouchers in India [54],
- Kenya’s Vouchers for Health programme [55–59],
- pilot voucher schemes in Pakistan [60,61],
- HealthyBaby vouchers in Uganda [62],
- Mekerere University Voucher programme in Uganda [63,64].

**Table 6:** Details of vouchers for maternity care services included in the systematic review.

Programme	Included studies, quality and sample size	Maternal and newborn health entitlements	Supply-side components	Period of programme implementation	Details of any changes to programme design	Source of funding
Pilot programme, Bangladesh	Rob *et al*. (2009)—low quality: 436 and 414 women at baseline and follow-up during 2007 and 2008 [49]	Vouchers were targeted to poor women, distributed free of charge and entitled the holder to 3 x ANC, childbirth services, 1 x PNC for mother and newborn, and transport costs for each service	Payments to healthcare providers for care	2007–2008	None identified	International non-governmental organisation (Population Council)
Maternal Health Voucher Scheme, Bangladesh	Ahmed and Khan (2011)—medium quality: 3,600 women during 2008 [46]; Hatt *et al*. (2010)—medium quality: 2,208 women during 2009 [47]; Nguyen *et al*. (2012)—medium quality: 2,208 women during 2009 [48]	Vouchers are targeted to poor women or distributed universally (depending on the district), are distributed free of charge and entitle the holder to 3 x ANC, FB or SBA at home, 1 x PNC for mother and newborn, CS, and transport costs, a gift box and cash for women who give birth in a facility	Payments to healthcare providers for care	2007-present	None identified	National government
Voucher programme, Cambodia	Van de Poel *et al*. (2014)—medium quality: 18,754 women during 2010 [50]	Vouchers were targeted to poor women or distributed universally (depending on the district), free of charge and entitled the holder to ANC, FB and PNC for mother and newborn at government healthcare facilities	Payments to healthcare providers for care	2007–2010	None identified	National government
Chiranjeevi Yojana, India	Bhat *et al*. (2009)—low quality: 656 women during 2006 [65]; De Costa *et al*. (2014)—high quality: state-wide data during 2000–2010 [52]; Mohanan *et al*. (2014)—high quality: 12,081 women during 2007–2009 and 2010 [53]	Free maternity care services for women with a below poverty line card	Payments to healthcare providers for care	2005-present	Phased roll-out; Reimbursement rates for providers increased	State government
Sambhav scheme, India	IFPS Project (2012)—medium quality (economic study): one district [54]	Vouchers were targeted to poor women, distributed free of charge and entitled the holder to 3 x ANC, FB and PNC for mother and newborn at accredited private hospitals	Payments to healthcare providers for care	2007–2013	None identified	Bilateral donor (USAID)
Vouchers for Health, Kenya	Amendah *et al*. (2013)—medium quality: 627 women during 2006–2012 [55]; Bellows *et al*. (2012)—medium quality: 1,914 and 2,448 women at baseline and follow-up during 2006 and 2009 [56]; Obare *et al*. (2012)—medium quality: 2,527 women during 2010–2011 [58]; Obare *et al*. (2014)—medium quality: 2,933 and 3,094 women at baseline and follow-up during 2010–2011 and 2012 [57]; Watt *et al*. (2015)—low quality: 934 and 569 women at baseline and follow-up during 2010 and 2012 [59]	Vouchers are targeted to poor women, sold for 200 shillings (USD 2.50) and entitle the holder to 4 x ANC, FB (including CS and treatment of neonatal complications if necessary) and PNC for mother and newborn up to six weeks after childbirth	Payments to healthcare providers for care	2006-present	Pilot scheme managed by a parastatal organisation. Phased expansion and transfer to Ministry of Health control. Switched from commission-based to salaried voucher distributors	Early phases funded by bilateral donor (KfW, Germany). National government has begun to contribute
Pilot programmes, Pakistan	Agha (2011a)—medium quality: 2,018 and 2,033 women at baseline and follow-up during 2009 and 2010 [60]; Agha (2011b)—low quality: 681 and 742 women at baseline and follow-up during 2010 [61]	Voucher booklets were targeted to poor women, sold for 100 rupees (USD 1.20) and entitled the holder to 3 x ANC, FB, 1 x PNC for mother and newborn, CS	Payments to healthcare providers for care	Urban: 2008–2009; Rural: 2010	None identified	Bilateral donor (USAID)
HealthyBaby vouchers, Uganda	Reproductive Health Vouchers Evaluation Team (2012)—low quality: 2,443 and 2,895 at baseline and follow-up during 2008 and 2010–2011 [62]	Vouchers are targeted to poor women, sold for 3,000 shillings (USD 1.50) and entitle the holder to 4 x ANC, childbirth services and PNC	Payments to healthcare providers for care	2008-present	Maternal health vouchers added to an existing reproductive health voucher programme	Bilateral donor (KfW, Germany) and the World Bank
Makerere Voucher Programme, Uganda	Alfonso *et al*. (2015)—low quality (economic study) and medium quality (quantitative study): sample chosen from among 810,618 women during 2007–2011 [63]; Mayora *et al*. (2014)—low quality (economic study) [64]	Vouchers were distributed universally in intervention areas, were free of charge and entitled the holders to FB and transportation. PNC for mother and newborn was included if mother/newborn experienced complications	Payments to healthcare providers for care, and transport providers received fixed payments for the average distance to travel in the intervention area	2009–2011	Antenatal care and PN vouchers withdrawn after pilot (2009–2010) due to unexpectedly large demand for vouchers	International non-governmental organisation (Bill and Melinda Gates Foundation)

Notes: ANC—antenatal care, FB—birth in healthcare facilities, SBA—birth with a skilled birth attendant, PNC—postnatal care, CS—caesarean section in case of obstetric complications

The studies were of medium quality and typically used controlled before-and-after approaches to compare intervention and control areas, with data analysis including regression analyses to control for potential confounding. Weaknesses included small sample sizes, non-random sampling strategies, and risk of leakage across study groups.

#### Vouchers for maternity care services and antenatal care

The effect of vouchers on uptake of antenatal care was examined by 11 studies (Table A8 in S5 Appendix—a report by Hatt *et al*. [47] was excluded from this section as it reported the same research as Nguyen *et al*. [48]). A before-and-after study without controls (n = 850 women) of a pilot programme in Bangladesh found evidence of an increase (compared to baseline findings) in the proportion of women receiving any antenatal care and proportion of women receiving three or more antenatal check-ups [49]. Similarly, two cross-sectional studies (n = 3,600 women and n = 2,208 women) of Bangladesh’s Maternal Health Voucher Scheme reported increases in the proportion of women receiving any antenatal care in intervention areas (compared to control areas) and increases in the proportion of women receiving three or more antenatal check-ups in intervention areas and among voucher recipients (compared to non-recipients) [46,48]. A before-and-after study without controls (n = 5,338) of Uganda’s HealthyBaby vouchers found that the programme increased the proportion of women receiving four or more antenatal checks in intervention areas compared to baseline areas prior to voucher distribution [62].

One before-and-after study without controls (n = 4,362 women) of Kenya’s Vouchers for Health programme found that receipt of any antenatal care and of at least four antenatal contacts increased among recipients, but no evidence of an effect in intervention areas overall [56]. Similarly, a cross-sectional study of the same programme reported no differences in number of antenatal contacts women attended in intervention areas [58]. Findings from a pilot programme in Pakistan (n = 4,051 women) reported increased receipt of at least three antenatal check-ups among voucher recipients (compared to non-recipients) [60], however another similar study (n = 1,423 women) found evidence of increased antenatal care uptake among women in the second poorest quintile but not amongst women who were in the poorest quintile [61].

A controlled before-and-after study (n = 12,081 women) of the Chiranjeevi Yojana programme in India found no evidence of an increase in uptake of antenatal care [53], and a cross-sectional study (n = 18,754) of a pilot voucher programme in Cambodia found no increase in the proportion of women who received three or more antenatal check-ups [50].

**Fig 9:**
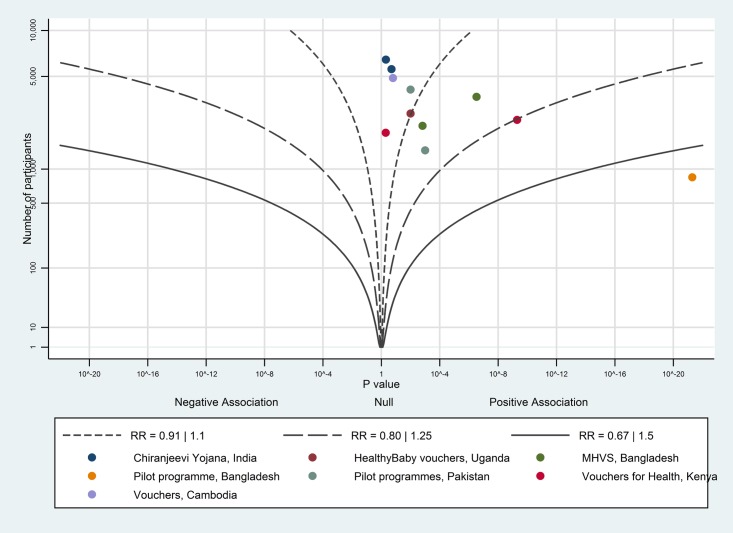
Albatross plot for effect of vouchers for maternity care services on uptake of antenatal care. MHVS—Maternal Health Voucher Scheme.

The albatross plot for the uptake of antenatal care is shown in Fig 9. All 11 data points show a positive association and most points fall around the RR effect contour of 1.1, corresponding to a 5 percentage point increase; we considered this to be a moderate effect.

#### Vouchers for maternity care services and birth with a skilled birth attendant

Five studies examined the effect of vouchers for maternity care services on birth with a skilled birth attendant (Table A9 in S5 Appendix—a report by Hatt *et al*. [47] was excluded from this section as it reported on the same data as Nguyen *et al*. [48]). A before-and-after study without controls (n = 850 women) found evidence that a pilot voucher scheme in Bangladesh increased births with a skilled birth attendant compared to baseline [49], and two cross-sectional studies (n = 3,600 women and n = 2,208 women) of Bangladesh’s Maternal Health Voucher Scheme found the programme increased births with a skilled birth attendant [46,48]. Bellows *et al*. [56] undertook a before-and-after study without controls (n = 4,362 women) of Kenya’s Vouchers for Health programme which found that the programme increased the proportion of women who had access to a skilled birth attendant in intervention areas and among voucher recipients. However a cross-sectional study (n = 2,527 women) which included data on women who had access to the same programme only found evidence of impact on birth with a skilled birth attendant in early intervention but not late-intervention areas [58].

A cross-sectional study (n = 3,600 women) of Bangladesh’s Maternal Health Voucher Scheme, using data from a district where voucher distribution was universal, indicated that births with a skilled birth attendant increased among the poorest and richest terciles, but inequity (measured as the ratio of uptake between the richest and the poorest terciles) was reduced [46].

**Fig 10:**
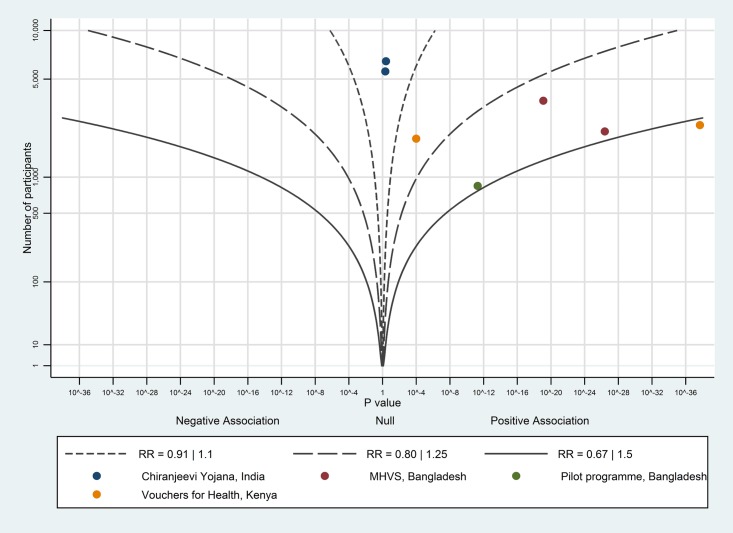
Albatross plot for effect of vouchers for maternity care services on births with a skilled birth attendant. MHVS—Maternal Health Voucher Scheme.

The albatross plot for births with a skilled birth attendant is shown in Fig 10. All seven studies showed a positive association; the magnitude of effect was likely more than a RR of 1.25, corresponding to a 12–13 percentage point increase; we considered this to be a large effect.

#### Vouchers for maternity care services and births in healthcare facilities

The impact of vouchers for maternity care services on proportions of births attended at healthcare facilities was examined by 13 studies (Table A10 in S5 Appendix—a report by Hatt *et al*. [47] and an article by Obare *et al*. [58] were excluded from this section as they reported the same research as Nguyen *et al*. [48], and Obare *et al*. [57], respectively). A before-and-after study without controls (n = 850 women) of a pilot voucher scheme in Bangladesh found an increase in births in healthcare facilities compared to baseline [49], and two cross-sectional studies (n = 3,600 women and n = 2,208 women) of Bangladesh’s Maternal Health Voucher Scheme found evidence of an increase compared to in intervention areas or among recipients [46,48]. Similarly, a cross-sectional study (n = 18,754) of a pilot voucher scheme in Cambodia found evidence of a positive effect on birth in healthcare facilities in intervention areas [50].

Three before-and-after studies (n = 6,027 women, n = 4,362 women and n = 627 women) found that the Vouchers for Health programme in Kenya increased the proportion of births in healthcare facilities in intervention areas, with women who received vouchers more likely to give birth in healthcare facilities in intervention areas [55–57]. Similarly, two before-and-after studies (n = 4,051 women and n = 1,423 women) of pilot voucher programmes in Pakistan also found births in healthcare facilities increased among recipients and more generally in intervention areas compared to areas assessed before roll out of the intervention [60,61]. A controlled before-and-after study (n = 5,338 women) determined that Uganda’s HealthyBaby vouchers increased births in healthcare facilities in intervention areas [62], and a retrospective area study (n = 810,618 women) of Uganda’s Makerere University Voucher Scheme found the programme increased births in participating hospitals [63]. No evidence of impact on births in healthcare facilities was found by studies of the Chiranjeevi Yojana [52,53].

Disaggregated data presented in a cross-sectional study of Bangladesh’s Maternal Health Voucher Scheme, using data from a district where voucher distribution was universal, indicated that the programme increased births in healthcare facilities among women in the poorest and in the richest terciles, but that inequity between the groups appeared smaller [46]. Similarly, a cross-sectional study of a pilot voucher programme in Cambodia found births in healthcare facilities increased among the poorest 40% of households regardless of whether voucher distribution was universal or targeted to the poorest households, but births in healthcare facilities did not increase among the other 60% of households [50]. Data from a before-and-after study on a pilot targeted voucher programme in Pakistan indicated that births in healthcare facilities increased for recipients of the vouchers, and disaggregated data showed increases for all wealth quintiles except the least poor [60]. A before-and-after study on a similar pilot voucher programme in another area in Pakistan found that births in healthcare facilities increased overall (p<0.001) and disaggregated data showed that the increase was limited to the poorest quintile [61].

**Fig 11:**
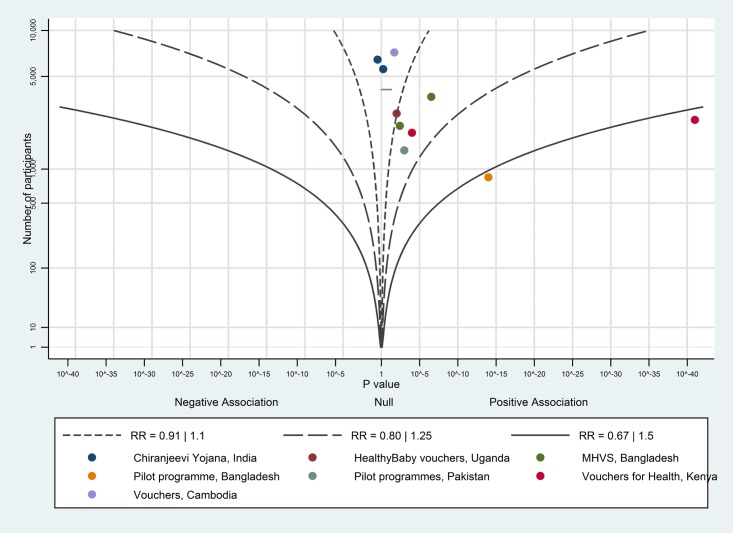
Albatross plot for effect of vouchers for maternity care services on births in a health facility. MHVS—Maternal Health Voucher Scheme.

The albatross plot for births in a healthcare facility is shown in Fig 11. Two studies [52,63] were not included in the albatross plot as the number of participants in each study was not available. Ten of the eleven studies showed a positive association; the magnitude of effect is likely to be a little above a RR of 1.1, corresponding to at least a 5 percentage point increase; we considered this to be a moderate effect.

#### Vouchers for maternity care services and birth with a skilled birth attendant or births in healthcare facilities in case of complications

Three studies examined the effect of vouchers for maternity care services on birth outcomes in cases of obstetric complication (a report by Hatt *et al*. [47] was excluded from this section as it reported the same research as Nguyen *et al*. [48]). The cross-sectional study (n = 3,600 women) by Ahmed and Khan [46] of Bangladesh’s Maternal Health Voucher Scheme found that odds of seeking medical assistance in case of obstetric complications among women who were voucher recipients was 1.5 (p<0.01) compared to non-recipients. Data disaggregated by wealth quintile indicated that the vouchers, which were distributed universally in the study district, increased treatment-seeking in case of obstetric complications for the richest tercile but not the poorest tercile.

A cross-sectional study (n = 2,208 women) by Nguyen *et al*. [48] found no evidence of effect (p>0.05) from the Maternal Health Voucher Scheme on caesarean section rate. Similarly, a cross-sectional study (n = 18,754) by van de Poel *et al*. [50] on a voucher programme in Cambodia found no differences in caesarean section rates compared to control areas.

#### Vouchers for maternity care services and postnatal care

Ten studies examined the effect of vouchers for maternity care services on postnatal care for mothers and newborns (Table A11 in S5 Appendix—a report by Hatt *et al*. [47] was excluded from this section as it reported the same research as Nguyen *et al*. [48]). Rob *et al*. [49] undertook a pilot study of a voucher scheme in Bangladesh which found that the programme increased the proportion of women and newborns receiving any postnatal care compared to baseline, and two cross-sectional studies (n = 3,600 women and n = 2,208 women) found that Bangladesh’s Maternal Health Voucher Scheme increased receipt of postnatal care for mothers and newborns among voucher recipients and in intervention areas [46,48]. Increased receipt of any postnatal care for mothers and newborns in intervention areas was also found in a study of Uganda’s HealthBaby vouchers which included data on 5,338 women [62], a similar finding to a cross-sectional study (n = 18,754) on a pilot voucher scheme in Cambodia [50].

A pilot voucher scheme in Pakistan which included data on 4,501 women found evidence of an increase in receipt of any postnatal care for mothers and newborns among voucher recipients (compared to non-recipients) [61], however a controlled before-and-after study (n = 1,423) found only a modest increase in postnatal care for the recipients of vouchers [60]. No evidence of effect on recipient of any postnatal care for mothers and newborns was found by studies on Chiranjeevi Yojana in India and Vouchers for Health in Kenya [53,58,65].

**Fig 12:**
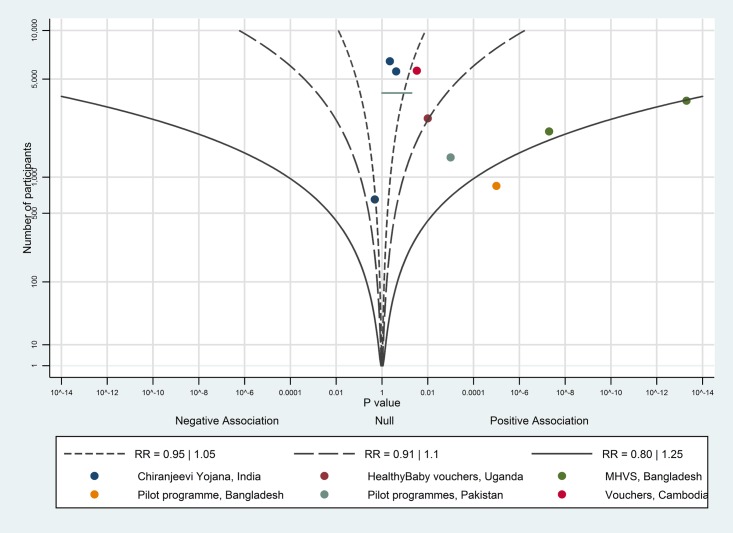
Albatross plot for effect of vouchers for maternity care services on uptake of postnatal care. MHVS—Maternal Health Voucher Scheme.

The albatross plot for uptake of postnatal care is shown in Fig 12. Nine of the ten studies showed a positive association; the magnitude of effect is likely to be a little above a RR of 1.1, corresponding to at least a 5 percentage point increase; we considered this to be a moderate effect.

#### Vouchers for maternity care services and quality of care

One study examined the effect of vouchers for maternity care services on quality of care [59]. Watt *et al*. scored postnatal care sessions (for women and newborns) against a checklist of services. The quality of care for mothers (including history-taking, physical examination, danger signs advice, family planning counselling, and risk assessment and management of HIV and sexually transmitted infections) improved by 86% (p<0.05) with no evidence of improvement (p>0.1) in quality of postnatal care for newborns (including feeding advice, newborn examination, danger signs advice and documentation).

#### Costs, cost-effectiveness and cost-utility of vouchers for maternity care services

Four studies examined the costs, cost-effectiveness or cost-utility of voucher for maternity care services (Table 7). A study of Bangladesh’s Maternal Health Voucher Scheme found that the incremental cost per birth with a skilled birth attendant was USD 69.85 [47]. An evaluation by the IFPS Technical Assistance Project [54] showed that the cost per childbirth voucher used in the Sambhav scheme in India was USD 66.60.

**Table 7:** Costs, cost-effectiveness and cost-utility of vouchers for maternity care services.

Study	Study data	Findings
*Maternal Health Voucher Scheme*, *Bangladesh (2007-present)*		
Hatt *et al*. (2010)	2009	Cost per voucher distributed: USD 41.37
		Incremental cost per birth with a skilled birth attendant: USD 69.85
*Sambhav scheme*, *India (2007–2013)*		
IFPS Technical Assistance Project (2012)	2011	Cost per antenatal care voucher used: USD 5.20
		Cost per delivery voucher used: USD 66.60
		Cost per postnatal care voucher used: USD 3.30
*Makerere University Voucher Scheme*, *Uganda (2009–2011)*		
Alfonso *et al*. (2015)	2007–2011	Cost per birth in a voucher hospital: USD 19.65
		Cost per birth in a voucher hospital (incl. ANC and PNC): USD 24.63
		Cost per DALY averted (societal perspective): USD 302
		Cost per DALY averted (medical sector perspective): USD 338
Mayora *et al*. (2014)	2010–2011	Cost per childbirth: USD 23.90
		Cost per postnatal care check-up: USD 7.90

Note. DALY denotes disability-adjusted life-year

Separate studies of the Makerere University Voucher Scheme in Uganda estimated the cost per childbirth to be USD 19.65 and USD 23.90 [63,64]. Alfonso *et al*. [63] calculated that the cost per disability-adjusted life year (DALY) averted was USD 302–338 depending on the perspective used for analysis.

### Vouchers for merit goods

Two studies examined the effect of the Tanzanian National Voucher Scheme (2004-present), a programme that offered vouchers which entitled users to a discount of 3,250 shillings (USD 2.70) off insecticide-treated nets [66,67]. The programme had a phased roll-out and the value of the discount increased over time. Sources of funding include the Global Fund to Fights AIDS, Tuberculosis and Malaria and the US President’s Malaria Initiative.

Hanson *et al*.’s [67] before-and-after study (sample size 6,199 women in 2005, 6,260 in 2006 and 6,198 in 2007) found that longer exposure to the programme was associated with increased use of insecticide-treated nets (p<0.1). Mulligan *et al*.’s [66] study found that the cost per insecticide-treated net delivered to a home was USD 7.57 and the cost per child’s death averted was USD 873.

## Discussion

This systematic review of effectiveness included 51 quantitative studies that presented evidence from 22 demand-side financing programmes across 20 countries in Latin America, sub-Saharan Africa and Asia. There are important distinctions between different types of demand-side financing, but there are now a sufficient number of studies (although only nine included studies were judged to be of high quality) from which to draw some conclusions about effects of such schemes on utilisation of maternity care services through the mechanisms of conditional cash transfers (16 studies), short-term cash payments (13 studies) or vouchers for maternity care services (19 studies).

There was some evidence that, with the exception of unconditional cash transfers, demand-side financing resulted in short- to medium-term positive effects on uptake of some (but not all) maternity care services (Table 8). There was limited evidence about a longer-term effect on uptake of maternity care services as most studies only collated data from a period of two to three years after programme introduction. Evaluations of the Chiranjeevi Yojana in India present a cautionary example in this regard. Early, methodologically poor studies suggested that the Chiranjeevi Yojana voucher-like scheme had successfully and significantly improved uptake of maternity care services. However, recent evaluations which used more robust study methods and collated data over a longer period found no evidence of effect.

**Table 8:** Summary of results from albatross plots for effects of demand-side financing on use of maternity care services.

	Antenatal care	Birth with a skilled birth attendant	Birth in a healthcare facility	Postnatal care
**Conditional cash transfers**	Large positive effect (estimated 10 percentage point increase)	Limited positive effect	Insufficient number of studies to draw conclusions	Insufficient number of studies to draw conclusions
**Unconditional cash transfers**	Insufficient number of studies to draw conclusions			
**Short-term cash payments to offset costs**	Limited positive effect	Small positive effect (estimated 2–3 percentage point increase)	Moderate positive effect (estimated 5 percentage point increase)	Limited negative effect, or no association
**Vouchers for maternity care services**	Moderate positive effect (estimated 5 percentage point increase)	Large positive effect (estimated 12–13 percentage point increase)	Moderate positive effect (estimated 5 percentage point increase)	Moderate positive effect (estimated 5 percentage point increase)
**Vouchers for merit goods**	Insufficient number of studies to draw conclusions			

Findings indicate that the effects of demand-side financing on uptake of maternity care services are programme- and context-specific. It is plausible that any improvements in the outcomes of interest may be more dependent on a complex set of factors including the baseline of healthcare and social infrastructure from which they start and the range of barriers and facilitators known to influence a woman’s ability to access good quality maternity care. The improvements that have been seen in the short- to medium-term uptake of maternity care services as a result of demand-side financing interventions appeared to be linked to the specific payment conditionalities or voucher entitlements.

The following general conclusions related to payment conditionalities can be drawn in this respect:

- conditional cash transfers that included among their conditionalities the uptake of antenatal care services appeared to have had an impact on the proportion of women receiving multiple antenatal check-ups, but findings were less clear with respect to receipt of any antenatal care, or on the uptake of other maternity care services in the continuum including childbirth and postnatal care (not included as conditionalities);
- the only published study identified on unconditional cash transfers found no difference in the uptake of any maternity care service;
- short-term cash payments which were contingent on the birth taking place in a facility or on having a skilled attendant at birth showed an increase on the uptake of these services, but findings were less clear of any effect on uptake of antenatal or postnatal care (even though these services are also typically included within voucher entitlements);
- vouchers for maternity care services increased the uptake of services for which they provided eligibility or subsidy, including antenatal care, skilled attendant at birth, facility births and, to a lesser extent, postnatal care;
- the only (non-economic) included study on vouchers for merit goods (in this case insecticide-treated nets) demonstrated an increase in the uptake of the nets.

Studies that disaggregated data by location found that demand-side financing increased uptake of maternity care services in urban and rural areas, and this may be determined by the perceived value of programme benefits relative to the costs for the family of accessing and receiving care.

Targeting and verification systems involve additional resource burdens but no studies were identified that had examined the costs of this aspect. Studies using disaggregated data found that even those demand-side financing programmes targeted at low-income households increased the uptake of services across multiple wealth quintiles, as did programmes without targeting. This suggests that in practice establishing and enforcing eligibility criteria may be difficult or ineffective, which is corroborated in a linked review of evidence on experiences with the implementation of cash transfers and vouchers [68]. Literature on cash transfers in other social sectors has suggested that clear eligibility criteria can be effective at targeting payments to the poorest groups [69,70], however payment conditionalities may be counter-productive. Studies on cash transfers in the education sector have indicated that conditionalities may make little difference to outcomes [71,72], and that conditionalities may systematically exclude specific vulnerable groups if they are less likely to fulfil programme requirements [73].

Studies that included sub-group analyses and presented data disaggregated by location or type of healthcare facility have also provided some (albeit weak) indications that demand-side financing programmes may be more effective at increasing uptake of maternity care services in areas with better quality healthcare facilities (defined as those offering a greater range of maternity care services) [42], and that effects on health outcomes are greater in areas with better health infrastructure [35]. Earlier systematic reviews similarly highlighted the importance of existing infrastructure and achieving at least a minimum level of quality for care [4,5,10].

Few studies (none of high quality) measured the effect of demand-side financing programmes on the quality of care received by women attending healthcare facilities. Those that did so used a narrow range of measures of ‘quality’ on which they found limited evidence of effect. Most indicators used to measure quality of care concerned receipt of specific procedures such as vaccinations or the provision of educational information on signs and symptoms of adverse health outcomes in pregnancy. While some studies found improvements on these specified indicators [21,24,39,43,59], others did not [31,39,43,59]. Only one study took a broader approach to quality of care that considered effects on hospital cleanliness, respectful behaviour by hospital staff and, conversely, use of dangerous practices [39]. That study found no differences in outcomes of interest.

It has been suggested that programmes including vouchers for maternity care services and output-based payments to providers are well placed to improve quality of care [74,75], however there remains a paucity of evidence to support that assertion. Only one study examined outcomes relevant to quality of care [59], and found that the quality of postpartum care for mothers improved (as assessed by the number of procedures performed including history-taking, physical examination, danger signs advice, family planning counselling, risk assessment and management of HIV and sexually transmitted infections) but no evidence of an effect on quality of postnatal care for newborns (when scored against a list of procedures including feeding advice, newborn examination, danger signs and symptoms advice and documentation). No studies considered ‘quality’ from the perspective of women service users.

The only published study so far to compare demand-side financing with supply-side investment (a short-term cash payment programme compared to free 24-hour care in a district hospital in the Indian state of Haryana) suggested that the latter may be equally as effective at increasing uptake of maternity care services [32], but further, robust evidence is required.

We identified a small number of studies which considered the cost-effectiveness of demand-side financing interventions. Where data on costs were considered, it was with regard to vouchers for maternity care services or merit goods and was largely limited to studies that determined costs per voucher used. The authors of a study on the Makerere University Voucher Scheme in Uganda [63] Êlculated an incremental cost-effectiveness ratio for the programme and noted that their result was highly cost-effective according to World Health Organization thresholds for health interventions. We found no comparative evidence to indicate if vouchers for maternity care services are more or less expensive than supply-side investment with similar effects on short-term coverage rates. Likewise, we found no comparative evidence on the cost-effectiveness of cash transfers with regards to use and quality of maternal and newborn health. Evaluative research on cash transfers in other social sectors suggests that such programmes have lower administrative costs than some supply-side investments, particularly in the medium- to long-term, but also that costs are increased by resource-intensive monitoring of conditionalities [76,77].

Review findings indicate a need for more methodologically robust studies that examine the effects of demand-side financing programmes in the longer term and not just during a pilot project or two-three years following programme launch, and for greater disaggregation of data according to wealth in order to determine the equity effects of programmes. There remains a paucity of research on effects of demand-side financing on maternal and infant health outcomes and women’s views of care received. Future studies need to incorporate multiple intervention arms in order to avoid research designs that only compare indicators in areas with a demand-side financing programme to those in areas with no additional investment at all. Without future research on programme cost-effectiveness and also on the extent of out-of-pocket costs incurred or defrayed by families as a result of participation in a demand-side financing programme, it will not be possible to determine whether demand-side financing approaches are a better policy option than increasing supply-side investments in public sector maternity provision.

This systematic review identified and retrieved evidence using a broad set of search terms and databases. One weakness of the review is that it only used English search terms and English language databases. The least methodologically robust studies (for example those that did not include comparators or statistical testing between groups) were excluded from the review however some of the included studies were at high risk of bias due to the observational design which lacked internal validity, small sample sizes and a lack of testing or adjustment for potential confounding factors.

Some evaluation studies compared recipients with non-recipients, a technique which may overestimate the impact of demand-side financing programmes if, in the absence of the programme, the recipients were already more likely to use maternity care services than non-recipients. This issue is particularly pertinent for programmes in which vouchers were sold to women (specifically the Vouchers for Health programme in Kenya, pilot voucher programmes in Pakistan and HealthyBaby vouchers in Uganda) as it is possible that women who purchased vouchers were already more inclined to seek maternity care and thus more likely to use them.

Finally, but perhaps most importantly, our comparisons between and within the different types of demand-side financing should be interpreted with caution due to the heterogeneity of programme aims, design, administration and setting. We have drawn conclusions based on evidence from 22 programmes in 20 countries, mostly conditional cash transfers, short-term cash payments and vouchers for maternity care services. There are common features, notably a transfer of resources to users of healthcare, but there are also important differences and we have drawn attention to specific issues that emerge from those differences throughout this paper.

## Conclusions

Demand-side financing approaches have been widely introduced in low- and middle-income countries during the last 10–15 years. Our systematic review consolidated evidence from seven published systematic reviews of evidence on the impact of different types of demand-side financing on maternal health, and updated the systematic searches to June 2015. A substantial number of studies have examined the effect of demand-side financing on the use of maternity care services, although many focus on an initial programme period, usually two or three years after initiation. Further evidence is still needed on whether programmes are effective longer-term. Findings suggest that demand-side financing approaches tied to service use (either via payment conditionalities or vouchers for selected services) could increase the uptake of specific maternity care services such as antenatal care, use of a skilled attendant at birth and in the case of vouchers, postnatal care, but that effects appear to be programme and context-specific and may depend on a complex set of barriers and facilitators related to social factors and conditions in the healthcare system. Effects were seen in rural and urban areas and across wealth quintiles. There are few studies to indicate that demand-side financing approaches have improved quality of maternity care or other maternal and newborn health outcomes. Comparative studies, including on whether these programmes are more cost-effective than supply-side investment in public services, research on social impact, including equity, research on health impact, including implications of increasing caesarean section rates, and cost-effectiveness studies are lacking.

## Supporting information

S1 Appendix Systematic review protocol. (DOCX)

S2 Appendix PRISMA checklist. (DOC)

S3 Appendix Critical appraisal results and bias assessment. (DOCX)

S4 Appendix Study characteristics tables. (DOCX)

S5 Appendix Supplementary results tables. (DOCX)
