# Supplementary material for: The effects of cash transfers and vouchers on the use and quality of maternity care services: A systematic review
Source: PLoS One. 2017 Mar 22;12(3):e0173068. doi: 10.1371/journal.pone.0173068 (PMC5362260; doi:10.1371/journal.pone.0173068)
Supplement: S1 Appendix — (DOCX) [file pone.0173068.s001.docx]

**S1 Appendix. Systematic review protocol.**

**Protocol for update of systematic review:** Demand-side financing measures to increase maternal health service utilisation and improve health outcomes: a systematic review of evidence from low- and middle-income countries

**Review question/objective**

The overall objective is to assess the effects of demand-side financing (DSF) interventions on maternal health service utilisation and on maternal and newborn health outcomes in low- and middle-income countries. Broader effects (of demand-side financing measures to increase maternal health service utilisation and improve maternal health outcomes) on the situation of perinatal and infant health, underprivileged women and the health care system will also be assessed.

**Specific review questions**

This review seeks to address the following questions with reference to DSF interventions that seek to improve maternal health:

What are the effects of different DSF interventions that promote maternal health on:

1. Uptake of maternal health services including antenatal care, birth with a skilled attendant, birth in a health facility, and postpartum care mother/postnatal care newborn?
2. Maternal morbidity and mortality?
3. Perinatal and infant morbidity and mortality?
4. The quality of care provided, including appropriate use of maternal and newborn health commodities including oxytocin, misoprostol, magnesium sulphate, injectable antibiotics, antenatal corticosteroid, chlorhexidine, and resuscitation equipment?
5. The choice of provider offered to consumers and competitiveness of the market?
6. The quality of life of expectant and new mothers?
7. Out-of-pocket expenditure and household poverty?
8. The responsiveness of providers (in terms of the scope of services, the number of providers and the way that services are provided)?

Can DSF measures provide a cost-effective approach to increase utilisation of maternal health services and improve health outcomes among rural, poor or socially excluded women?

**Inclusion criteria**

### Types of participants

The primary target group will be poor, rural or socially excluded women of all ages who are either pregnant or within 42 days of the conclusion of pregnancy, the limit for postpartum care as defined by the World Health Organization. Definitions for ‘poor’, ‘rural’ and ‘socially excluded’ vary between and within countries as they are relative terms founded in cultural and political contexts. Other groups, such as non-poor women, will be included to examine issues such as equity of uptake and leakage in DSF programmes. For the purposes of this review we will document the definitions employed by authors to describe target populations. Providers of services through DSF mechanisms will also be considered.

### Intervention/phenomenon of interest

The intervention of interest is any programme that incorporates DSF as a method to increase the consumption of goods and services that have an impact on maternal health outcomes. This includes the direct consumption of maternal healthcare goods and services as well as related ‘merit goods’ such as improved nutrition. Our review will not include conventional insurance systems as a form of DSF because they have been the subject of other reviews.

We will include systems in which potential users of maternal health services are financially empowered to make restricted decisions on buying maternal health-related goods or services (sometimes known as consumer-led demand-side financing). These typically take the form of conditional cash transfers, or of schemes in which prospective users are given vouchers, coupons or cards, sometimes in conjunction with a choice between providers. We will also include programmes that provide unconditional cash benefits to pregnant women (for example in the form of maternity allowances). We will not, however, include more general employment-related interventions such as statutory maternity or unemployment benefit, tax credits or rebates which are usually part of more general social security systems.

Context

### The review will only consider studies and articles concerned with populations in low- and middle-income countries as defined by the World Bank. Inclusion will be based on the income status of a country at the time when the study was undertaken.

### Types of outcomes

*Quantitative*

The quantitative outcomes used in research on this topic vary with study design. Primary outcomes will be indicators to measure the utilisation of maternity care services, including antenatal, childbirth and postpartum care mother/postnatal care newborn, using terms such as coverage, uptake and access. It is likely that many studies on utilisation of maternal health services have used the proportion of births at a health facility or births with a skilled birth attendant present. Evidence that measures the effect of DSF on caesarean section rates will also be included.

Other outcomes including maternal mortality and morbidity and perinatal and neonatal mortality and morbidity will also be included.

Secondary outcomes for the review will include changes in the number and responsiveness of health service providers, changes in out-of-pocket expenditure, for example as a proportion of household income, and changes in household poverty, such as the proportion of households in the bottom quintile. Measures of quality of care (including use of life-saving commodities) and quality of life for pregnant and postnatal women will also be included.

*Economic*

### Outcomes measures relating to the unit costs of DSF programmes or to cost-effectiveness, (such as cost per institutional delivery) and to cost-utility (such as per quality- or disability-adjusted life year gained) will be included in the review.

### Types of studies/publications

### In order to be considered for inclusion studies must involve some comparison, such as more than one group, or a point in time, such as baseline data.

*Quantitative*

Randomised controlled trials will be sought for inclusion in the review but, in the absence of these, other experimental and non-experimental study designs may be included. Study designs that minimise the potential for bias will be given preference in accordance with the following hierarchy:

1. Experimental studies – randomised controlled trials, non-randomised controlled trials, quasi-experimental designs, and before and after studies.
2. Observational studies – prospective and retrospective cohort studies, case control studies, and analytical cross-sectional studies.

*Economic*

Retrospective and prospective studies of cost-effectiveness, cost-utility and unit cost will be sought for inclusion in the review.

*Qualitative*

Qualitative studies will be recorded and maintained for future work to better understand implementation factors and values and preferences of key stakeholders.

## Search strategy

The search strategy aims to find both published and unpublished studies. A search using a matrix of keywords and index terms will be undertaken across all included databases. The reference list of all identified reports and articles will be searched for additional studies.

We will consider studies published since 1990. DSF measures have been incorporated into maternal health strategies in low- and middle-income countries during the last 15 years. Our review seeks to include studies arising during this period of time and discussions of the DSF approach in the years preceding this period. An earlier version of the review (by Murray *et al.*) conducted searches for 1990 to June 2012. We will extend the earlier study by conducting searches for July 2012 to Dec 2014.

The databases and e-journal services to be searched include:

Applied Social Sciences Index and Abstracts,

ArticleFirst,

British Development Library Services,

EBSCO Host (includes CINAHL and MEDLINE),

Cochrane Central Register of Controlled Trials,

EconLit,

Electronic Collections Online,

HealthSource: Nursing/Academic Edition,

International Bibliography of the Social Sciences,

Latin-American and Caribbean Center on Health Sciences Information (LILACS),

Sage Journals Online,

ScienceDirect,

SCOPUS,

Social Policy and Practice,

Social Services Abstracts,

Sociological Abstracts,

SpringerLink,

Web of Knowledge, and

Wiley Online Library.

The search for unpublished studies and papers will include:

Archives of relevant governmental and non-governmental organisations and development banks,

Intute,

Nexis UK,

Mednar,

ProQuest Dissertations and Theses,

Qual Page,

Scirus, and

WorldWideScience.org.

Keywords to be used for our search will be:

“Abortion”

“Antenatal”

“Birth”

“Cash transfer”

“Child benefit”

“Cost”

“Cost-effective”

“Cost-utility”

“Demand side financing”

“Demand-side financing”

“Family allowance”

“Food stamps”

“Health service utilisation”

“Incentive”

“Infant”

“Maternal”

“Maternity allowance”

“Maternity benefit”

“Midwifery”

“Monetary transfer”

“Neonatal”

“Morbidity”

“Mortality”

“Obstetric”

“Output-based aid”

“Perinatal”

“Postpartum”

“Postnatal”

“Pregnancy”

“Reimbursement mechanism”

“Results-based financing”

“Voucher”

## Assessment of methodological quality

Papers selected for retrieval will be assessed by two independent reviewers for methodological validity prior to inclusion in the review. This will be done using standardised critical appraisal instruments from the Joanna Briggs Institute (see Appendix I). These are:

- the Joanna Briggs Institute Meta Analysis of Statistics Assessment and Review Instrument (JBI-MAStARI) for quantitative papers, and
- the Joanna Briggs Institute Analysis of Cost, Technology and Utilisation Assessment and Review Instrument (JBI-ACTUARI) for economic data

Any disagreements that arise between the reviewers will be resolved through discussion, or with a third reviewer.

## Data collection

Quantitative

Quantitative data will be extracted by two independent reviewers from papers included in the review using the standardised data extraction tool from JBI-MAStARI (see Appendix II). The data extracted will include specific details about the interventions, populations, study methods, programme theory and outcomes of significance to the review question and specific objectives.

Economic

## Economic data will be extracted by two independent reviewers from papers included in the review using the standardised data extraction tool from JBI-ACTUARI (see Appendix II). The data extracted will include specific details about the interventions, populations, cost, currency, study methods, programme theory and outcomes of significance to the review question and specific objectives.

## Data synthesis

Quantitative

Quantitative papers will, where possible, be pooled in statistical meta-analysis. Where statistical pooling is not possible, the findings will be presented in narrative form including tables and figures to aid in data presentation where appropriate. Findings from descriptive studies will, where possible, be synthesised and presented in a tabular summary with the aid of narrative and figures where appropriate.

Economic

Economic findings will, where possible, be pooled and presented in a tabular summary. Where this is not possible, findings will be presented in narrative form.

**Appendix I: JBI Critical appraisal forms**

**Appendix II: JBI Data extraction forms**
