# Supplementary material for: The effects of cash transfers and vouchers on the use and quality of maternity care services: A systematic review
Source: PLoS One. 2017 Mar 22;12(3):e0173068. doi: 10.1371/journal.pone.0173068 (PMC5362260; doi:10.1371/journal.pone.0173068)
Supplement: S3 Appendix — (DOCX) [file pone.0173068.s003.docx]

**S3 Appendix. Critical appraisal results and bias assessment**

| **Studies with data on health outcomes, service use and/or quality of care** | | | | | | | | | | |
| --- | --- | --- | --- | --- | --- | --- | --- | --- | --- | --- |
| **Author, year** | **1) Is sample representative of patients in the population as a whole?** | **2) Are the patients at a similar point in the course of their condition/illness?** | **3) Has bias been minimised in relation to selection of cases and of controls?** | **4) Are confounding factors identified and strategies to deal with them stated?** | **5) Are outcomes assessed using objective criteria?** | **6) Was follow up carried out over a sufficient time period?** | **7) Were the outcomes of people who withdrew described and included in the analysis?** | **8) Were outcomes measured in a reliable way?** | **9) Was appropriate statistical analysis used?** | **Overall study quality (low, medium or high) and possible bias** |
| **Conditional cash transfers** | | | | | | | | | | |
| **Bolsa Familia, Brazil** | | | | | | | | | | |
| Guanais (2013) | Y | Y | Y | Y | Y | Y | N | Y | Y | High Large variation between regions |
| Shei (2013) | Y | Y | Y | Y | Y | Y | N | Y | Y | High |
| **Comunidades Solidarias Rurales, El Salvador** | | | | | | | | | | |
| De Brauw *et al.* (2011) | Y | Y | Y | Y | Y | N | N | Y | Y | Medium Short time between surveys Small sample sizes Concurrent increases in service availability |
| **Mi Familia Progresa, Guatemala** | | | | | | | | | | |
| Gutierrez *et al.* (2011) | Y | Y | Y | Y | Y | N | N | Y | Y | Medium Short follow-up |
| **Programa de Asignación Familia, Honduras** | | | | | | | | | | |
| Morris *et al.* (2004) | Y | Y | Y | N | Y | N | N | Y | Y | Medium Short follow-up  Results not verified by government data |
| **Program Keluarga Harapan, Indonesia** | | | | | | | | | | |
| Alatas *et al.* (2011) | Y | Y | Y | Y | Y | N | N | Y | Y | Medium  Short follow-up Some areas allocated to the programme did not implement it, and vice versa |
| Triyana (2012) | Y | Y | Y | Y | Y | N | N | Y | Y | Medium  Short follow-up Some areas allocated to the programme did not implement it, and vice versa |
| **Prospera, Mexico** | | | | | | | | | | |
| Barber and Gertler (2008) | Y | Y | Y | Y | Y | N | N | Y | Y | Medium  Short follow-up Risk of recall bias |
| Barber and Gertler (2009) | Y | Y | Y | Y | Y | N | N | Y | Y | Medium  Short follow-up Quality defined as receipt of specific procedures |
| Barber (2010) | Y | Y | Y | Y | Y | N | N | Y | Y | Medium  Short follow-up Rate of CS remained <15% despite increase |
| Barham (2011) | Y | Y | Y | Y | Y | Y | N | Y | Y | High Possible weaknesses in reporting |
| Hernandez Prado *et al.* (2004a) | Y | Y | Y | Y | Y | Y | N | Y | Y | High Risk of underlying confounders |
| Hernandez Prado *et al.* (2004b) | Y | Y | U | Y | Y | Y | N | Y | Y | Medium Wide variation of results within study |
| Sosa-Rubai *et al.* (2011) | Y | Y | Y | Y | Y | Y | N | Y | Y | High Possible confounding effect of another programme |
| Urquieta *et al.* (2009) | Y | Y | U | Y | Y | N | Y | Y | Y | Medium Short follow-up |
| **Plan de Atención Nacional a la Emergencia Social (PANES), Uruguay** | | | | | | | | | | |
| Amarante *et al.* (2011) | Y | Y | Y | Y | Y | N | N | Y | Y | Medium |
| **Unconditional cash transfers** | | | | | | | | | | |
| **Child Grant Programme, Zambia** | | | | | | | | | | |
| Handa *et al.* (2015) | Y | Y | Y | Y | Y | N | N | Y | Y | Medium  Short follow-up |
| **Short-term cash payments to offset costs of access** | | | | | | | | | | |
| **CHIMACA project, China** | | | | | | | | | | |
| Hemminki *et al.* (2013) | Y | Y | Y | Y | Y | N | N | Y | Y | Medium  Short follow-up Small sample size |
| **Janani Suraksha Yojana, India** | | | | | | | | | | |
| Amudhan *et al.* (2013) | U | Y | Y | N | Y | Y | N | Y | Y | Medium Possible crossover between intervention and control groups Role of underlying trends |
| Carvalho *et al.* (2014) | Y | Y | Y | Y | Y | N | N | Y | Y | Medium  Short follow-up  Includes non-JSY payments |
| Joshi and Sivaram (2014) | Y | Y | Y | Y | Y | N | N | Y | Y | Medium  Short follow-up |
| Lim *et al.* (2010) | Y | Y | Y | Y | Y | N | N | Y | Y | Medium  Short follow-up Includes non-JSY payments |
| Mazumdar *et al.* (2012) | U | Y | U | Y | Y | N | N | Y | Y | Medium  Short follow-up |
| Purohit *et al.* (2014) | U | Y | Y | N | Y | Y | N | Y | Y | Low Small sample size Risk of underlying confounders / secular trends |
| Randive *et al.* (2013) | Y | Y | Y | Y | Y | N | N | Y | Y | Medium Short follow-up  Risk of underlying confounders / secular trends |
| Santhya *et al.* (2011) | U | Y | N | Y | Y | Y | N | Y | Y | Medium Respondent selection methods (specifically which areas were chosen) is unclear |
| Vora *et al.* (2012) | Y | Y | Y | Y | Y | N | N | Y | Y | Medium  Short follow-up Includes non-JSY payments |
| **Safe Delivery Incentive Programme, Nepal** | | | | | | | | | | |
| Powell-Jackson *et al.* (2009) | Y | Y | U | Y | Y | N | N | Y | Y | Medium  Short follow-up |
| Powell-Jackson and Hanson (2012) | Y | Y | Y | Y | Y | N | N | Y | Y | Medium  Short follow-up Risk of recall bias |
| **SURE-P, Nigeria** | | | | | | | | | | |
| Okoli *et al.* (2014) | Y | Y | Y | Y | Y | N | N | Y | Y | Medium  Short follow-up Catchment area used as denominator Non-random selection of intervention areas |
| **Vouchers for maternity care services** | | | | | | | | | | |
| **Pilot voucher programme, Bangladesh** | | | | | | | | | | |
| Rob *et al.* (2009) | Y | Y | U | N | Y | N | N | Y | Y | Low  Short follow-up No control areas Risk of underlying confounders / secular trends |
| **Maternal Health Voucher Scheme, Bangladesh** | | | | | | | | | | |
| Ahmed and Khan (2011) | Y | Y | Y | Y | Y | N | N | Y | Y | Medium  Short follow-up  No baseline study  Pre-existing differences between intervention and control areas |
| Hatt *et al.* (2010) | Y | Y | Y | Y | Y | N | N | Y | Y | Medium  Short follow-up Questionable power to detect mortality |
| Nguyen *et al.* (2012) | Y | Y | Y | Y | Y | N | N | Y | Y | Medium  Short follow-up No indication of voucher uptake |
| **Voucher programme, Cambodia** | | | | | | | | | | |
| Van de Poel *et al.* (2014) | Y | Y | Y | Y | Y | N | N | Y | Y | Medium  Short follow-up Risk of recall bias |
| **Chiranjeevi Yojana, India** | | | | | | | | | | |
| Bhat *et al.* (2009) | Y | Y | N | N | N | N | N | Y | Y | Low Single time point used Risk of underlying confounders / differences between intervention and non-intervention arms  Short follow-up |
| De Costa *et al.* (2014) | Y | Y | Y | Y | Y | Y | N | Y | Y | High |
| Mohanan *et al.* (2014) | Y | Y | Y | Y | Y | Y | N | Y | Y | High Risk of recall bias |
| **Vouchers for Health, Kenya** | | | | | | | | | | |
| Amendah *et al.* (2013) | Y | Y | Y | Y | Y | Y | N | Y | Y | Medium Small sample sizes |
| Bellows *et al.* (2012) | U | Y | U | Y | Y | N | N | Y | Y | Medium  Short follow-up No control areas |
| Obare *et al.* (2012) | Y | Y | N | Y | Y | N | N | Y | Y | Medium  Short follow-up Leakage of vouchers to non-poor women Respondents selected within 5 kilometres of a health facility |
| Obare *et al.* (2014) | Y | Y | Y | Y | Y | Y | N | Y | Y | Medium  Leakage of vouchers to non-poor women Respondents selected within 5 kilometres of a health facility |
| Watt *et al.* (2015) | U | Y | U | Y | Y | Y | N | U | Y | Low Not clear how respondents were selected Unclear who the data collector was or whether they had an effect on results |
| **Pilot programmes, Pakistan** | | | | | | | | | | |
| Agha (2011b) | Y | Y | N | Y | Y | N | N | Y | Y | Low No control areas Short follow-up |
| Agha (2011a) | Y | Y | Y | Y | Y | N | N | Y | Y | Medium  Short follow-up  Purposive selection of intervention areas |
| **HealthyBaby vouchers, Uganda** | | | | | | | | | | |
| Reproductive Health Vouchers Evaluation Team (2012) | Y | Y | Y | N | Y | Y | N | Y | Y | Low No control areas Risk of underlying confounders / secular trends |
| **Makerere University Voucher Scheme, Uganda** | | | | | | | | | | |
| Alfonso *et al.* (2015) | U | Y | N | Y | Y | Y | N | Y | Y | Medium Baseline coverage based on household survey but effect estimates based on data in registers Non-random selection of intervention districts |
| **Vouchers for merit goods** | | | | | | | | | | |
| **Tanzanian National Voucher Scheme, Tanzania** | | | | | | | | | | |
| Hanson *et al.* (2009) | Y | Y | Y | Y | Y | Y | N | Y | Y | High Concurrent ITN distribution programmes |

| **Studies with data on costs and cost-effectiveness** | | | | | | | | | | | | |
| --- | --- | --- | --- | --- | --- | --- | --- | --- | --- | --- | --- | --- |
| **Author, year** | **1) Is there a well-defined question?** | **2) Is there a comprehensive description of alternatives?** | **3) Are all important and relevant costs and outcomes for each alternative identified?** | **4) Has clinical effectiveness been established?** | **5) Are costs and outcomes measured accurately?** | **6) Are costs and outcomes valued credibly?** | **7) Are costs and outcomes adjusted for differential timing?** | **8) Is there an incremental analysis of costs and consequences?** | **9) Were sensitivity analyses conducted to investigate uncertainty in estimates of cost or consequences?** | **10) Do study results include all issues of concerns to users?** | **11) Are the results generalisable to the setting of interest in the review?** | **Overall study quality (low, medium or high) and possible bias** |
| **Vouchers for maternity care services** | | | | | | | | | | | | |
| **Maternal Health Voucher Scheme, Bangladesh** | | | | | | | | | | | | |
| Hatt *et al.* (2010) | U | N | Y | U | Y | Y | U | U | N | N | Y | Medium Cost to patients not included No comparator |
| **Sambhav scheme, India** | | | | | | | | | | | | |
| IFPS Technical Assistance Project (2012) | N | N | Y | N | Y | Y | U | N | N | U | Y | Medium No comparator |
| **Makerere University Voucher Scheme, Uganda** | | | | | | | | | | | | |
| Alfonso *et al.* (2015) | Y | Y | Y | N | Y | Y | Y | Y | Y | Y | N | Low Effectiveness data at risk of bias Programme costs substantially lower than those report by Mayora *et al.*, which uses similar data |
| Mayora *et al.* (2014) | Y | N | Y | N/A | Y | Y | Y | Y | N | N | Y | Low Programme costs substantially higher than those report by Alfonso *et al.*, which uses similar data No calculation of whether increased inputs would be required and therefore increase costs Referral transport costs not included |
| **Vouchers for merit goods** | | | | | | | | | | | | |
| **Tanzanian National Voucher Scheme, Tanzania** | | | | | | | | | | | | |
| Mulligan *et al.* (2008) | Y | N | N | Y | Y | Y | Y | Y | Y | Y | U | High Used established guidelines for measuring costs and published estimates of effectiveness |
