# Supplementary material for: The effects of cash transfers and vouchers on the use and quality of maternity care services: A systematic review
Source: PLoS One. 2017 Mar 22;12(3):e0173068. doi: 10.1371/journal.pone.0173068 (PMC5362260; doi:10.1371/journal.pone.0173068)
Supplement: S4 Appendix — (DOCX) [file pone.0173068.s004.docx]

**S4 Appendix. Study characteristics tables**

| **Study** | **Methods** | **Participants** | **Intervention** | **Context** | **Study size** | **Relevant outcomes** |
| --- | --- | --- | --- | --- | --- | --- |
| Agha (2011) | Household surveys and bivariate analyses | Randomly selected women who had given birth in the 12 months preceding the survey | Pilot voucher scheme (vouchers for maternity care services) | Dera Ghazi Khan City (urban), Pakistan | Baseline: 681  Follow-up: 742 | 3 x ANC, FB, any PNC |
| Agha (2011) | Household survey and bivariate analyses | Randomly selected women who had given birth in the 12 months preceding each survey | Pilot voucher scheme (vouchers for maternity care services) | Jhang district (rural), Pakistan | Baseline: 2,018  Follow-up: 2,033 | 3 x ANC, FB, any PNC |
| Ahmed and Khan (2011) | Household survey and logistic regression analyses | Women who had given birth within a year prior to the survey | Maternal Health Voucher Scheme (vouchers for maternity care services) | Sarishabari district (predominantly rural), Bangladesh | 3,600 | 3 x ANC, SBA, FB, any PNC |
| Alatas *et al.* (2011) | Household survey and difference-in-difference regression analyses | Married women aged 16-49 years | Program Keluarga Harapan (conditional cash transfers) | Rural and urban districts in Indonesia | Baseline: 14,987  Follow-up: 14,922 | 4 x ANC, average ANC, SBA, FB, average PNC, 2 x PNC, neonatal mortality, infant mortality |
| Alfonso *et al.* (2015) | Healthcare facility registers and difference-in-difference regression analyses. Secondary sources for cost data | Participating hospitals and women living in programme areas | Makerere University Voucher Scheme (vouchers for maternity care services) | Kamuli and Pallisa districts, Uganda | Sample chosen from among 810,618 women | FB, cost per birth, cost per DALY averted |
| Amarante *et al.* (2011) | Household survey and surveillance data, and difference-in-difference analyses | Women living in eligible households | Plan de Atención Nacional a la Emergencia Social (PANES) (conditional cash transfers) | Uruguay | 67,863 | Perinatal morbidity, maternal morbidity, total ANC, SBA |
| Amendah *et al.* (2013) | Household surveys and regression analyses | Women with at least two children who had been pregnant while living in a slum | Vouchers for Health (vouchers for maternity care services) | Two urban slums in Nairobi, Kenya | 627 | FB for subsequent pregnancy |
| Amudhan *et al.* (2013) | Community surveillance data and difference-in-difference analyses | Women who gave birth at a government facility in the surveillance area | Janani Suraksha Yojana (payments to offset costs of access) | Rural area in Haryana, India | 7,796 | FB |
| Barber and Gertler (2008) | Household surveys and regression analyses | Poor women aged 15-49 in poor rural communities | Prospera (conditional cash transfers) | Rural communities, Mexico | 840 | Perinatal morbidity, any ANC, average ANC, 5 x ANC |
| Barber and Gertler (2009) | Household surveys and regression analyses | Poor women aged 15-49 in poor rural communities | Prospera (conditional cash transfers) | Rural communities, Mexico | 892 | QoC |
| Barber (2010) | Household surveys and regression analyses | Poor women aged 15-49 in poor rural communities | Prospera (conditional cash transfers) | Rural communities, Mexico | 979 | CS |
| Barham (2011) | Household surveys and regression analyses | Poor women aged 15-49 in poor rural communities | Prospera (conditional cash transfers) | Rural communities, Mexico | 19,421 | Infant mortality, neonatal mortality |
| Bellows *et al.* (2012) | Household surveys and logistic regression analyses | Females aged 12–54 years who had given birth during the two years preceding the surveys | Vouchers for Health (vouchers for maternity care services) | Informal settlements in Nairobi (urban), Kenya | Baseline: 1,914  Follow-up: 2,448 | Any ANC, 4 x ANC, SBA, FB |
| Bhat *et al.* (2009) | Household survey and bivariate analyses | Women in households selected from an intervention district | Chiranjeevi Scheme (vouchers for maternity care services) | Dahod district (mixed urban and rural), India | 656 | Any PN |
| Carvalho *et al.* (2014) | Household surveys and logistic regression analyses | Ever-married women aged 15-44 | Janani Suraksha Yojana (payments to offset costs of access) | National sample of districts (mixed urban and rural), India | 23,924 | Any PN, any PP |
| de Brauw and Peterman (2011) | Household surveys and regression discontinuity analyses | Households with children under 3 years old or a pregnant woman | Comunidades Solidarias Rurales (conditional cash transfers) | Rural communities, El Salvador | Baseline: 269  Follow-up: 287 | 5 x ANC, SBA, FB, any PNC |
| De Costa *et al.* (2014) | Community surveillance data, household surveys and census data. Analyses were conducted using piece-wise regression | Women who gave birth in a healthcare facility | Chiranjeevi Scheme (vouchers for maternity care services) | Gujarat, India | Not stated, approximately 10-12 million | FB (CS not included as no significance testing) |
| Guanais (2013) | Household survey and least squares regression | Women aged 15-45 | Bolsa Familia (conditional cash transfers) | Brazil | 54,253 | Post-neonatal mortality |
| Gutierrez *et al.* (2011) | Household surveys and regression analyses | Women living in eligible households | Mi Familia Progresa (conditional cash transfers) | Guatemala | 4,563 households | Average ANC |
| Handa *et al.* (2015) | Household survey and difference-in-difference analyses | Women who gave birth at least 15 months before the survey | Child Grant Programme (conditional cash transfers) | Three rural districts in Zambia | Baseline:1,155  Follow-up: 559 | 4 x ANC, QoC, SBA |
| Hanson *et al.* (2009) | Household surveys and regression analyses | Women aged 15-49 | Tanzanian National Voucher Scheme (vouchers for merit goods) | 21 districts (mixed urban and rural), Tanzania | 2005: 6,199  2006: 6,260  2007: 6,198 | Ownership and use of insecticide-treated nets |
| Hatt *et al.* (2010) | Household survey, programme expenditure data and probit regression analyses | Eligible women who had delivered in the six months preceding the survey, representatives of the Ministry of Health and Family Welfare, WHO and DFID | Maternal Health Voucher Scheme (vouchers for maternity care services) | Early implementation subdistricts (mainly rural), Bangladesh | 2,208 | 3 x ANC, FB, CS, any PN, QoC, cost per voucher distributed, incremental cost per birth with a skilled attendant |
| Hemminki *et al.* (2013) | Household surveys and logistic regression analyses | Pregnant women who used maternity care | CHIMACA project (payments to offset costs of access) | One county in Anhui province, China | 592 | 5 x ANC, CS, any PN, QoC |
| Hernandez Prado *et al.* (2004a) | Household surveys and mortality databases. Analysis was done using linear mixed models | Municipalities with one or more household enrolled in Prospera | Prospera (conditional cash transfers) | Rural, semi-urban and urban areas, Mexico | 2,445 municipalities | Maternal mortality, infant mortality |
| Hernandez Prado *et al.* (2004b) | Household surveys and regression analyses | All poor women aged 15-49 eligible to be incorporated into Prospera | Prospera (conditional cash transfers) | Rural, semi-urban and urban areas, Mexico | 1998-2000: 29,041  2003: 7,802 | Any ANC, average ANC, 5 x ANC, SBA, FB, QoC |
| IFPS Technical Assistance Project (2012) | Interviews, focus group discussions, survey data and programme budgets | Health service administrators, voucher management agency, community leaders, health workers and women who received vouchers | Sambhav voucher scheme (vouchers for maternity care services) | Haridwar district in Uttarakhand, India | One district  246 interviews with participants | Cost per voucher used |
| Joshi and Sivaram (2014) | Household surveys and difference-in-difference analyses | Ever-married women aged 15-44 who gave less than three years before the survey | Janani Suraksha Yojana (payments to offset costs of access) | National sample of districts (mixed urban and rural), India | 425,708 | 3 x ANC, SBA, any PN |
| Lim *et al.* (2010) | Household surveys and multivariate regression models using matching, with-versus-without comparison, and differences-in-difference analyses | Ever-married women aged 15-44 | Janani Suraksha Yojana (payments to offset costs of access) | National sample of districts (mixed urban and rural), India | 2002-2004: not stated  2007-2009: 182,869 | 3 x ANC, SBA, FB, perinatal mortality, neonatal mortality |
| Mayora *et al.* (2014) | Programme administrative records and basic statistics | Participating hospitals | Makerere University Voucher Scheme (vouchers for maternity care services) | Kamuli and Pallisa districts, Uganda | 810,618 | Cost per additional delivery, cost per additional PN |
| Mazumdar *et al.* (2012) | Household surveys and difference-in-difference models | Ever-married women aged 15-44 | Janani Suraksha Yojana (payments to offset costs of access) | National sample of districts (mixed urban and rural), India | 344,903 | 3 x ANC, SBA, FB, neonatal mortality |
| Mohanan *et al.* (2014) | Household survey and difference-in-difference analyses | Women who had given birth within the previous 5 years | Chiranjeevi Scheme (vouchers for maternity care services) | Gujarat, India | 12,081 | Any ANC, FB, any PN |
| Morris *et al.* (2004) | Household surveys and mixed effects regression analyses | Pregnant women and mothers of children younger than 3 years old | Programa de Asignación Familia (conditional cash transfers) | Rural municipalities, Honduras | 11,002 households | 5 x ANC, any PN |
| Mulligan *et al.* (2008) | Budgets and survey data, semi-structured interviews and basic statistics | Project staff, providers and women who used the vouchers | Tanzania National Voucher Scheme (voucher for merit goods) | Tanzania | National programme | Cost per insecticide-treated net distributed |
| Nguyen *et al*. (2012) | Household survey and probit and linear regression analyses (with difference-in-difference analyses) | Women who gave birth in the 6 months preceding the survey | Maternal Health Voucher Scheme (vouchers for maternity care services) | Early implementation subdistricts (mainly rural), Bangladesh | 2,208 | 3 x ANC, SBA, FB, any PN |
| Obare *et al.* (2012) | Household survey and analyses using Chi-square tests and logit models | Women aged 15-49 who gave birth in the 12 months preceding the survey (or was pregnant at the time of the survey) | Vouchers for Health (vouchers for maternity care services) | Six districts (all mixed urban and rural), Kenya | 2,527 | 4 x ANC, SBA, FB, any PN |
| Obare *et al.* (2014) | Household survey and analyses using Chi-square tests and logit models | Women aged 15-49 who gave birth in the 12 months preceding the survey (or was pregnant at the time of the survey) | Vouchers for Health (vouchers for maternity care services) | Six districts (all mixed urban and rural), Kenya | Baseline: 2,933  Follow-up: 3,094 | FB |
| Okoli *et al.* (2014) | Programme surveillance data and regression analyses | Women living in rural and underserved areas | SURE-P (payments to offset costs of access) | Nine states, Nigeria | 2,022,801 | Any ANC, 4 x ANC, SBA |
| Powell-Jackson *et al.* (2009) | Household survey, propensity score matching and regression analyses | Every woman who gave birth in the study district | Safe Delivery Incentive Programme (payments to offset costs of access) | Makwanpur district (rural), Nepal | 14,799 | SBA, FB, neonatal mortality |
| Powell-Jackson and Hanson (2012) | Community surveillance data and regression analyses | Women who had given birth during the 3 years before the survey | Safe Delivery Incentive Programme (payments to offset costs of access) | Six districts (representing mountains, hills, and flat terrains) in Nepal | 5,901 | SBA, FB, CS |
| Purohit *et al.* (2014) | Household survey and chi-squared analyses | Women who had given birth within the last year | Janani Suraksha Yojana (payments to offset costs of access) | Four districts in Rajasthan, India | 424 | 3 x ANC, any PN, QoC |
| Randive *et al.* (2013) | Community surveillance data, household surveys and multiple regression analyses | Women who gave birth (surveillance data) and ever married women aged 15-49 who gave birth less than two years prior to surveys | Janani Suraksha Yojana (payments to offset costs of access) | 284 districts across nine Indian states | Not stated | FB |
| Reproductive Health Vouchers Evaluation Team (2012) | Household surveys and bivariate analyses | Women aged 15-49 years who had a pregnancy or birth during the 12 months preceding the survey and men whose partner was eligible | HealthyBaby vouchers (vouchers for maternity care services) | 6 districts, Uganda | Baseline: 2,443  Follow-up: 2,895 | 4 x ANC, FB, any PN, personal expenditure |
| Rob *et al.* (2009) | Cross-sectional surveys and bivariate analyses | Poor women who had given birth in the 12 months preceding each survey (endline included only recipients) | Pilot voucher scheme (vouchers for maternity care services) | Habiganj district (rural), Bangladesh | Baseline: 436  Follow-up: 414 | 3 x ANC, SBA, FB, any PN |
| Santhya *et al.* (2011) | Household survey data and propensity score matching. Analyses performed using bivariate significance testing and difference-in-difference models | Women aged below 35 years who had given birth in the 12 months preceding the survey | Janani Suraksha Yojana (payments to offset costs of access) | Alwar and Jodhpur districts (mixed rural and urban), India | 4,770 | 3 x ANC, SBA, FB, any PN, QoC |
| Shei (2013) | Community surveillance data and regression analyses | Neonates, post-neonates and infants | Bolsa Familia (conditional cash transfers) | Brazil | Not stated – national data | Neonatal mortality, post-neonatal mortality, infant mortality |
| Sosa-Rubai *et al.* (2011) | Household surveys and logit and probit regression models | Women aged 15-49 years, with at least one child aged less than 24 months, and living in rural areas | Prospera (conditional cash transfers) | Rural communities, Mexico | 5,051 | Average ANC |
| Triyana (2014) | Household surveys and multivariate analyses | Married women aged 16-49, midwives, health centres | Program Keluarga Harapan (conditional cash transfers) | Rural and urban areas in Indonesia | Baseline: 14,987  Follow-up: 14,922 | Infant mortality, maternal mortality, neonatal morbidity, SBA, QoC |
| Urquieta *et al.* (2009) | Household surveys and analyses using regression discontinuity analysis and difference-in-difference models | Women aged 15-49 years in poor rural communities | Prospera (conditional cash transfers) | Rural communities, Mexico | 2,790 | SBA |
| van de Poel *et al.* (2014) | Household surveys and difference-in-difference analyses | Women of reproductive age who gave birth during the previous 5 years | Voucher programme (vouchers for maternity care services) | Nationally representative sample, Cambodia | 18,754 | 3 x ANC, FB, any PN |
| Vora *et al.* (2012) | Household survey and logistic regression analyses | Poor ever married women aged 15-49 years | Janani Suraksha Yojana (payments to offset costs of access) | Rural areas in Gujarat and Tamil Nadu, India | 2,267 | 3 x ANC, FB, CS |
| Watt *et al.* (2015) | Observations of provider-patient interactions and difference-in-difference analyses | Women attending postnatal care at a randomly selected accredited hospital | Vouchers for Health (vouchers for maternity care services) | One hospital in Kenya | Baseline: 934  Follow-up: 569 | QoC |

Notes. ANC denotes antenatal care, FB birth in a healthcare facility, SBA birth with a skilled attendant, PN postnatal care, PP postpartum care, CS caesarean section in case of obstetric complication, QoC quality of care
