# Supplementary material for: The effects of cash transfers and vouchers on the use and quality of maternity care services: A systematic review
Source: PLoS One. 2017 Mar 22;12(3):e0173068. doi: 10.1371/journal.pone.0173068 (PMC5362260; doi:10.1371/journal.pone.0173068)
Supplement: S5 Appendix — (DOCX) [file pone.0173068.s005.docx]

**S5 Appendix. Supplementary results tables**

| **Table A1. Effect of conditional cash transfers on uptake of antenatal care** | | | |
| --- | --- | --- | --- |
| **Study** | **Study data** | **Effect** | **95% confidence interval, standard error or p-value** |
| *Comunidades Solidarias Rurales, El Salvador (2005-present)* |  |  |  |
| De Brauw and Peterman (2011) | 2008 | No evidence of effect^4^ | p>0.1 |
| *Mi Familia Progresa, Guatemala (2008-present)* |  |  |  |
| Gutierrez *et al.* (2011) | 2009, 2010 | 11.0 percentage point increase^2^ | p<0.05 |
| *Programa de Asignación Familia, Honduras (1990-present)* |  |  |  |
| Morris *et al.* (2004) | 2000, 2002 | 18.7% increase in intervention areas^4^ | p<0.05 |
| *Program Keluarga Harapan, Indonesia (2008-present)* |  |  |  |
| Alatas *et al.* (2011) | 2007, 2009 | Increased by 0.5 visits^2^  7.2 percentage point increase^3^ | se: 0.2 (p<0.05)  se: 0.0 (p<0.01) |
| *Prospera, Mexico (1997-present)* |  |  |  |
| Hernandez Prado *et al.* (2004) | 2003 | No evidence of effect in early intervention rural areas^1^  6.7% increase in late intervention rural areas^1^  No evidence of effect in urban areas^1^  11.9% increase in early intervention rural areas^2^  No evidence of effect in late rural areas^2^  No evidence of effect in urban areas^2^  24.6% increase in early rural areas^4^  27.9% increase in late rural areas^4^ | p>0.05  p<0.05  p>0.1  p<0.05  p>0.1  p>0.1  p<0.05  p<0.01 |
| Barber and Gertler (2008) | 2003 | No evidence of effect^1^  No evidence of effect^2^  No evidence of effect^4^ | p>0.1  p>0.1  p>0.1 |
| Sosa-Rubâi *et al.* (2011) | 2007 | No evidence of effect^2^ | p>0.1 |
| *Plan de Atención Nacional a la Emergencia Social, Uruguay (2005-2007)* |  |  |  |
| Amarante *et al.* (2011) | 2003-2007 | 2.2% increase among recipients^2^ | p<0.05 |

Notes. ^1^ any antenatal care, ^2^ mean number of visits, ^3^ four or more antenatal visits, ^4^ five or more antenatal visits

| **Table A2. Effect of conditional cash transfers on birth with a skilled birth attendant** | | | |
| --- | --- | --- | --- |
| **Study** | **Study data** | **Effect** | **95% confidence interval, standard error or p-value** |
| *Comunidades Solidarias Rurales, El Salvador (2005-present)* |  |  |  |
| De Brauw and Peterman (2011) | 2008 | Range of results depending on regression model, including  No evidence of effect  17.4 percentage point increase | se: 9.7 (p>0.1)  se: 5.7 (p<0.01) |
| *Program Keluarga Harapan, Indonesia (2008-present)* |  |  |  |
| Alatas *et al.* (2011) | 2007, 2009 | No evidence of effect | p>0.05 |
| Triyana (2014) | 2007, 2009 | 9.2 percentage point increase | se: 4.9 (p<0.1) |
| *Prospera, Mexico (1997-present)* |  |  |  |
| Hernandez Prado *et al.* (2004) | 1998-2000, 2003 | No evidence of effect in early intervention rural areas  20.1% increase in late intervention rural areas  10.9-11.3% decrease in urban areas | p>0.1  p<0.05  p<0.05 |
| Urquieta *et al.* (2009) | 1998, 2000 | No evidence of effect | p>0.1 |
| Sosa-Rubai *et al.* (2011) | 2007 | OR: 2.4 in early intervention areas  OR: 3.3 in late intervention areas | se: 0.9 (p<0.05)  se: 1.4 (p<0.01) |
| *Plan de Atención Nacional a la Emergencia Social, Uruguay (2005-2007)* |  |  |  |
| Amarante *et al.* (2011) | 2003-2007 | No evidence of effect | p>0.1 |

| **Table A3. Effect of short-term cash payments on uptake of antenatal care** | | | |
| --- | --- | --- | --- |
| **Study** | **Study data** | **Effect** | **95% confidence interval, standard error or p-value** |
| *CHIMACA, China (2007-2009)* |  |  |  |
| Hemminki *et al.* (2013) | 2007-2009 | No evidence of effect^5^ | p>0.05 |
| *Janani Suraksha Yojana, India (2006-present)* |  |  |  |
| Joshi and Sivaram (2014) | 2002-2004, 2007-2009 | No evidence of effect^3^ | p>0.1 |
| Lim *et al.* (2010) | 2002-2004, 2007-2009 | 10.7-11.1% increased probability among recipients^3^  10.4-11.1% increased probability among recipients in high-focus states^3^  3.2-4.7% increased probability among recipients in non-high-focus states^3^ | CI: 4.6, 17.2  CI: 9.2, 12.2  CI: 1.6, 5.6 |
| Mazumdar *et al.* (2012) | 2002-2004, 2007-2009 | 3.9 percentage point increase^3^ | se: 2.0 (p<0.1) |
| Purohit *et al.* (2014) | 2011 | 10.0 percentage points higher among recipients^3^ | p<0.05 |
| Santhya *et al.* (2011) | 2009, 2010 | OR: 2.2 among recipients^3^  7% increase using difference-in-difference analyses^3^ | p<0.05  p<0.05 |
| Vora (2012) | 2007-2009 | No evidence of effect^3^ | p>0.1 |
| *Safe Delivery Incentive Programme, Nepal (2005-present)* |  |  |  |
| Powell-Jackson *et al.* (2009) | 2001-2007 | 3.1 percentage point increase^2^ | p<0.05 |
| *SURE-P, Nigeria (2013-present)* |  |  |  |
| Okoli *et al.* (2014) | 2013-2014 | No evidence of effect^1^  Decrease in proportion in intervention areas^4^  Increase in positive trend in intervention areas^4^ | p>0.05  p<0.05  p<0.01 |

Notes. ^1^ any antenatal care, ^2^ mean number of visits, ^3^ three or more antenatal visits, ^4^ four or more antenatal visits, ^5^ five or more antenatal visits

| **Table A4. Effect of short-term cash payments on births with a skilled birth attendant** | | | |
| --- | --- | --- | --- |
| **Study** | **Study data** | **Effect** | **95% confidence interval, standard error or p-value** |
| *Janani Suraksha Yojana, India (2006-present)* |  |  |  |
| Lim *et al.* (2010) | 2002-2004, 2007-2009 | 36.2-39.3% increased probability among recipients  58.7% increased probability among recipients in high-focus states  4.9% increased probability among recipients in non-high-focus states | CI: 33.7, 45.0  CI: 58.0, 59.5  CI: 3.6, 6.3 |
| Joshi and Sivaram (2014) | 2002-2004, 2007-2009 | 2.9 percentage point increase among eligible women | p<0.01 |
| Mazumdar *et al.* (2012) | 2002-2004, 2007-2009 | 8.1 percentage point increase from baseline | se: 1.8 (p<0.01) |
| Santhya *et al.* (2011) | 2009, 2010 | Mean difference: 49.0 percentage points higher among recipients | p<0.001 |
| *Safe Delivery Incentive Programme, Nepal (2005-present)* |  |  |  |
| Powell-Jackson *et al.* (2009) | 2001-2007 | 2.3 percentage point increase | p<0.05 |
| Powell-Jackson and Hanson (2012) | 2008 | 16.6% increase compared to controls | p<0.05 |
| *SURE-P, Nigeria (2013-present)* |  |  |  |
| Okoli *et al.* (2014) | 2013-2014 | No evidence of effect | p>0.05 |

| **Table A5. Effect of short-term cash payments on birth taking place at a healthcare facility** | | | |
| --- | --- | --- | --- |
| **Study** | **Study data** | **Effect** | **95% confidence interval, standard error or p-value** |
| *Janani Suraksha Yojana, India (2006-present)* |  |  |  |
| Amudhan *et al.* (2013) | 2006-2010 | OR: 1.7-1.8 | p<0.001 |
| Lim *et al.* (2010) | 2002-2004, 2007-2009 | 43.5-49.2% increased probability among recipients  63.8-64.5% increased probability among recipients in high-focus states  6.6-8.0% increased probability among recipients in non-high-focus states | CI: 33.7, 45.0  CI: 63.0, 65.2  CI: 4.9, 9.0 |
| Mazumdar *et al.* (2012) | 2002-2004, 2007-2009 | 12.0 percentage point increase  22.0 percentage point increase in high-focus states  6.8 percentage point increase in non-high-focus states | se: 1.9 (p<0.01)  se: 2.2 (p<0.01)  se: 4.7 (p>0.1) |
| Randive *et al.* (2013) | 2005-2010, 2010-2011 | 29.0 percentage point increase | p<0.05 |
| Santhya *et al.* (2011) | 2009, 2010 | Mean difference: 66 percentage points higher among recipients | p<0.001 |
| Vora (2012) | 2007-2009 | OR: 3.9 in one state (Gujarat)  No evidence of effect in one state (Tamil Nadu) | p<0.05  p>0.1 |
| *Safe Delivery Incentive Programme, Nepal (2005-present)* |  |  |  |
| Powell-Jackson *et al.* (2009) | 2001-2007 | No evidence of effect | p>0.05 |
| Powell-Jackson and Hanson (2012) | 2008 | 17.8% increase compared to controls | p<0.05 |

| **Table A6. Effect of short-term cash payments on caesarean section rates** | | | |
| --- | --- | --- | --- |
| **Study** | **Study data** | **Effect** | **95% confidence interval, standard error or p-value** |
| *CHIMACA, China (2007-2009)* |  |  |  |
| Hemminki *et al.* (2013) | 2007-2009 | OR: 1.8 | p<0.05 |
| *Janani Suraksha Yojana, India (2006-present)* |  |  |  |
| Mazumdar *et al.* (2012) | 2002-2004, 2007-2009 | No evidence of effect | p>0.1 |
| Vora *et al.* (2012) | 2007-2009 | No evidence of effect in one state (Gujarat)  OR: 1.7 in one state (Tamil Nadu) | p>0.1  p<0.05 |
| *Safe Delivery Incentive Programme, Nepal (2005-present)* |  |  |  |
| Powell-Jackson *et al.* (2009) | 2001-2007 | No evidence of effect | p>0.05 |
| Powell-Jackson and Hanson (2012) | 2008 | 35.5% increase in caesarean sections compared to women who did not know about the programme before giving birth  23.9% increase in assisted deliveries (including caesarean sections) compared to women who did not know about the programme before giving birth | p<0.1  p<0.05 |

| **Table A7. Effect of short-term cash payments on postnatal care for mothers and newborns** | | | |
| --- | --- | --- | --- |
| **Study** | **Study data** | **Effect** | **95% confidence interval, standard error or p-value** |
| *CHIMACA, China (2007-2009)* |  |  |  |
| Hemminki *et al.* (2013) | 2007-2009 | No evidence of effect | p>0.05 |
| *Janani Suraksha Yojana, India (2006-present)* |  |  |  |
| Carvalho *et al.* (2014) | 2007-2009 | 25.7 percentage point increase | p<0.05 |
| Joshi and Sivaram (2014) | 2002-2004, 2007-2009 | 19.2 percentage point decrease | p<0.01 |
| Purohit *et al.* (2014) | 2011 | 20.6 percentage points increase | p<0.01 |

| **Table A8. Effect of vouchers for maternity care services on uptake of antenatal care** | | | |
| --- | --- | --- | --- |
| **Study** | **Study data** | **Effect** | **95% confidence interval, standard error or p-value** |
| *Maternal Health Voucher Scheme, Bangladesh (2007-present)* |  |  |  |
| Ahmed and Khan (2011) | 2008 | OR: 1.9 among recipients^2^ | se: 0.13 (p<0.001) |
| Nguyen *et al.* (2012) | 2009 | 17.4 percentage points higher probability than in control areas^1^  24.1 percentage points higher probability than in control areas ^2^ | se: 3.3 (p<0.001)  se: 7.6 (p<0.001) |
| *Pilot voucher scheme, Bangladesh (2007-2008)* |  |  |  |
| Rob *et al.* (2009) | 2007, 2008 | 9.8 percentage point increase from baseline^1^  33.2 percentage point increase from baseline^2^ | p<0.01  p<0.01 |
| *Pilot voucher scheme, Cambodia (2007-2010)* |  |  |  |
| van de Poel *et al.* (2014) | 2010 | No evidence of effect^2^ | p>0.1 |
| *Chiranjeevi Yojana, India (2005-present)* |  |  |  |
| Mohanan *et al.* (2014) | 2007-2009, 2010 | No evidence of effect^1^ | p>0.1 |
| *Vouchers for Health, Kenya (2006-present)* |  |  |  |
| Bellows *et al.* (2012) | 2006, 2009 | No evidence of effect in intervention areas^1^  OR: 16.5 among recipients^1^  OR: 0.7 in intervention areas^3^  OR: 1.9 among recipients^3^ | CI: 0.5, 1.0  CI: 4.0, 68.1  CI: 0.6, 0.8  CI: 1.6, 2.4 |
| Obare *et al.* (2012) | 2010 | No evidence of effect in early intervention areas^3^  No evidence of effect in late intervention areas^3^ | CI: 0.8, 1.6  CI: 0.7, 1.4 |
| *Pilot voucher scheme, Pakistan (2008-2010)* |  |  |  |
| Agha (2011a) | 2009, 2010 | OR: 1.6 among recipients^2^ | p<0.01 |
| Agha (2011b) | 2010 | OR: 5.0 among recipients^2^ | p<0.001 |
|  |  |  |  |
| *HealthyBaby Vouchers, Uganda (2008-present)* |  |  |  |
| Reproductive Health Vouchers Evaluation Team (2012) | 2008, 2010-2011 | 10.0 percentage point increase in intervention areas^3^ | p<0.01 |

Notes. ^1^ any antenatal care, ^2^ three or more antenatal visits, ^3^ four or more antenatal visits

| **Table A9. Effect of vouchers for maternity care services on birth with a skilled birth attendant** | | | |
| --- | --- | --- | --- |
| **Study** | **Study data** | **Effect** | **95% confidence interval, standard error or p-value** |
| *Maternal Health Voucher Scheme, Bangladesh (2007-present)* |  |  |  |
| Ahmed and Khan (2011) | 2008 | OR: 3.6 among recipients | se: 0.1 |
| Nguyen *et al.* (2012) | 2009 | 46.4 percentage points higher probability than in control areas | se: 4.3 (p<0.001) |
| *Pilot voucher scheme, Bangladesh (2007-2008)* |  |  |  |
| Rob *et al.* (2009) | 2007, 2008 | 16.1 percentage point increase from baseline | p<0.01 |
| *Vouchers for Health, Kenya (2006-present)* |  |  |  |
| Obare *et al.* (2012) | 2010-2011 | OR: 2.0 in early intervention areas  No evidence of effect in late intervention areas | CI: 1.4, 2.8  p>0.05 |
| Bellows *et al.* (2012) | 2006, 2009 | OR: 1.2 in intervention areas  OR: 12.9 among recipients | CI: 1.0, 1.4  CI: 8.9, 19.3 |

| **Table A10. Effect of vouchers for maternity care services on births attended at healthcare facilities** | | | |
| --- | --- | --- | --- |
| **Study** | **Study data** | **Effect** | **95% confidence interval, standard error or p-value** |
| *Maternal Health Voucher Scheme, Bangladesh (2007-present)* |  |  |  |
| Ahmed and Khan (2011) | 2008 | OR: 2.5 among recipients | se: 0.18 |
| Nguyen *et al.* (2012) | 2009 | 13.6 percentage points higher probability than in control areas | se: 4.7 (p<0.001) |
| *Pilot voucher scheme, Bangladesh (2007-2008)* |  |  |  |
| Rob *et al.* (2009) | 2007, 2008 | 16.1 percentage point increase from baseline | p<0.01 |
| *Pilot voucher scheme, Cambodia (2007-2010)* |  |  |  |
| Van de Poel *et al.* (2014) | 2010 | 10.1 percentage point increase in intervention areas | p<0.05 |
| *Chiranjeevi Yojana, India (2005-present)* |  |  |  |
| De Costa *et al.* (2014) | 2000-2010 | No evidence of effect | p>0.1 |
| Mohanan *et al.* (2014) | 2007-2009, 2010 | No evidence of effect | p>0.1 |
| *Vouchers for Health, Kenya (2006-present)* |  |  |  |
| Obare *et al.* (2014) | 2010-2011, 2012 | OR: 2.0 in early intervention areas  OR: 1.7 in late intervention areas | CI: 1.4, 2.8  CI: 1.2, 2.4 |
| Bellows *et al.* (2012) | 2006, 2009 | OR: 1.4 in intervention areas  OR: 14.5 among recipients | CI: 1.2, 1.6  CI: 10.0, 21.7 |
| Amendah *et al.* (2013) | 2006-2012 | OR: 3.9 for subsequent birth for women who received a voucher, yet OR: 4.7 for subsequent birth for women who did not receive a voucher | p<0.05  p<0.01 |
| *Pilot voucher programmes, Pakistan (2008-2010)* |  |  |  |
| Agha (2011a) | 2009, 2010 | OR: 1.5 among recipients | p>0.05 |
| Agha (2011b) | 2010 | OR: 4.0 among recipients | p<0.001 |
| *HealthyBaby vouchers, Uganda (2008-present)* |  |  |  |
| Reproductive Health Vouchers Evaluation Team (2012) | 2008, 2010-2011 | 9.0 percentage point increase in intervention areas | p<0.01 |
| *Mekerere University Vouchers, Uganda* |  |  |  |
| Alfonso *et al.* (2015) | 2007-2011 | 52.3 percentage point increase in demand in participating hospitals (of which 9.4 percentage points were new users) | p<0.001 |

| **Table A11. Effect of vouchers for maternity care services on receipt of any postnatal care for mothers and newborns** | | | |
| --- | --- | --- | --- |
| **Study** | **Study data** | **Effect** | **95% confidence interval, standard error or p-value** |
| *Maternal Health Voucher Scheme, Bangladesh (2007-present)* |  |  |  |
| Ahmed and Khan (2011) | 2008 | OR: 2.7 among recipients | se: 0.14 |
| Nguyen *et al.* (2012) | 2009 | 19.1 percentage points higher probability than in control areas | se: 3.5 (p<0.001) |
| *Pilot voucher scheme, Bangladesh (2007-2008)* |  |  |  |
| Rob *et al.* (2009) | 2007, 2008 | 14.9 percentage point increase | p<0.01 |
| *Pilot voucher scheme, Cambodia (2007-2010)* |  |  |  |
| van de Poel *et al.* (2014) | 2010 | 5.3 percentage point increase in intervention areas | p<0.05 |
| *Chiranjeevi Yojana, India (2005-present)* |  |  |  |
| Mohanan *et al.* (2014) | 2007-2009, 2010 | No evidence of effect | p>0.1 |
| Bhat *et al.* (2009) | 2006 | No evidence of effect | p>0.05 |
| *Vouchers for Health, Kenya (2006-present)* |  |  |  |
| Obare *et al.* (2012) | 2010 | No evidence of effect in early intervention areas  No evidence of effect in late intervention areas | CI: 0.9, 1.8  CI: 0.6, 1.2 |
| *Pilot voucher scheme, Pakistan (2008-2010)* |  |  |  |
| Agha (2011b) | 2010 | OR: 5.8 among recipients | p<0.001 |
| Agha (2011a) | 2009, 2010 | OR: 1.3 among recipients | p>0.05 |
| *HealthyBaby vouchers, Uganda (2008-present)* |  |  |  |
| Reproductive Health Vouchers Evaluation Team (2012) | 2008, 2010-2011 | 8.0 percentage point increase in intervention areas | p<0.01 |
